# Oscillating PDF in termini of circadian pacemaker neurons and synchronous molecular clocks in downstream neurons are not sufficient for sustenance of activity rhythms in constant darkness

**DOI:** 10.1371/journal.pone.0175073

**Published:** 2017-05-30

**Authors:** Pavitra Prakash, Aishwarya Nambiar, Vasu Sheeba

**Affiliations:** 1 Evolutionary and Integrative Biology Unit, Jawaharlal Nehru Centre for Advanced Scientific Research, Bangalore, India; 2 Neuroscience Unit, Jawaharlal Nehru Centre for Advanced Scientific Research, Bangalore, India; McGill University, CANADA

## Abstract

In *Drosophila*, neuropeptide Pigment Dispersing Factor (PDF) is expressed in small and large ventral Lateral Neurons (sLNv and lLNv), among which sLNv are critical for activity rhythms in constant darkness. Studies show that this is mediated by rhythmic accumulation and likely secretion of PDF from sLNv dorsal projections, which in turn synchronises molecular oscillations in downstream circadian neurons. Using targeted expression of a neurodegenerative protein Huntingtin in LNv, we evoke a selective loss of neuropeptide PDF and clock protein PERIOD from sLNv soma. However, PDF is not lost from sLNv dorsal projections and lLNv. These flies are behaviourally arrhythmic in constant darkness despite persistence of PDF oscillations in sLNv dorsal projections and synchronous PERIOD oscillations in downstream circadian neurons. We find that PDF oscillations in sLNv dorsal projections are not sufficient for sustenance of activity rhythms in constant darkness and this is suggestive of an additional component that is possibly dependent on sLNv molecular clock and PDF in sLNv soma. Additionally, despite loss of PERIOD in sLNv, their activity rhythms entrain to light/dark cycles indicating that sLNv molecular clocks are not necessary for entrainment. Under constant light, these flies lack PDF from both soma and dorsal projections of sLNv, and when subjected to light/dark cycles, show morning and evening anticipation and accurately phased morning and evening peaks. Thus, under light/dark cycles, PDF in sLNv is not necessary for morning anticipation.

## Introduction

*Drosophila melanogaster* exhibit locomotor activity rhythms under 12h light: 12h dark cycles (LD), with a morning bout close to lights-ON and an evening bout close to lights-OFF and relative inactivity in the middle of the day and night [1]. This rhythm persists with a circadian period (~ 24h) even under constant darkness (DD). About 150 neurons in the adult fly brain comprise the circadian circuit that modulates various aspects of activity rhythm and are divided into subgroups of Lateral Neurons (LN: Ventral Lateral Neurons, LNv and Dorsal Lateral Neurons, LNd) and Dorsal Neurons (DN: DN1a, DN1p, DN2 and DN3) [2]. Circadian oscillations in the levels of PERIOD (PER) and TIMELESS (TIM) proteins are central components of the molecular clock in these neurons [3]. Functional circadian clocks in LNv, specifically small LNv are known to be necessary and sufficient for behavioural rhythmicity in DD [4,5]. Neuropeptide Pigment Dispersing Factor (PDF) expressed in 8 LNv per hemisphere: 4 small LNv (sLNv) and 4–5 large LNv (lLNv) controls rhythmic locomotor activity in DD [6,7]. Flies lacking PDF are similar to flies lacking LNv: mostly arrhythmic and the few weakly rhythmic flies have short period [7]. PDF receptor (PDFR) mutants phenocopy *pdf*^*01*^ and PDF acting via PDFR on LNv and other circadian neurons is sufficient to rescue rhythmicity [8–11]. PDF in the sLNv dorsal projections (DP) accumulates rhythmically and is possibly secreted rhythmically [12]. In the absence of external time cues, PDF is known to act as a coupling signal that synchronises the molecular clocks among circadian neurons to bring about coherent locomotor activity rhythms [12–20]. In the absence of PDF, in DD, molecular oscillations in the sLNv subgroup dampen and become asynchronous, while oscillations in other circadian neurons (LNd, DN1 and DN2) become decoupled from sLNv and run with a short period reflected as weak activity rhythms of short period [7,13,14,17,20]. Additional studies suggest complex functions for PDF—drives molecular rhythms in some neuronal groups, synchronises molecular oscillations among certain circadian neuronal groups and shortens or lengthens period in other neuronal groups [15,21–23]. Studies show that loss of behavioural rhythmicity occurs with disruption of PDF oscillations in sLNv DP, suggesting that PDF oscillations are necessary for activity rhythms in DD [16,17,24]. In the above studies, the molecular clock in sLNv is intact, and it is the output i.e. PDF oscillations that are affected. However, it is not known whether upon disruption of the sLNv molecular clock, PDF oscillation in sLNv DP and its downstream synchronising functions are affected. Also, it is unknown whether in the absence of PDF in sLNv soma, oscillating PDF in sLNv DP can sustain rhythmic activity. We therefore set out to assess the role of sLNv circadian molecular clock and PDF in modulating locomotor activity rhythms in DD and asked whether this function of PDF is restricted to its oscillations in sLNv DP.

Using the neurodegenerative polyglutamine protein Huntingtin (Glutamine repeat number>35), we achieved selective loss of PDF from sLNv soma, while not affecting PDF in lLNv [25,26]. In such flies, we assessed the role of PDF in sLNv and its oscillations in DP in driving activity rhythms in DD. We find that these flies lack PDF and PER in sLNv soma but show PDF oscillations in sLNv DP and synchronous PER oscillations in downstream circadian neurons, yet are behaviourally arrhythmic in DD. This suggests that PDF oscillations in sLNv DP are not sufficient for activity rhythms in DD. Our results are also indicative of an additional component in sLNv for sustenance of activity rhythms in DD that is independent of PDF oscillations in sLNv DP and possibly dependent on PDF in sLNv soma and PER-driven molecular clock in sLNv.

Under LD, flies without PDF or lacking PDF expressing LNv or with downregulated PDF do not have morning (M) peak and have a phase advanced evening (E) peak [7,27]. PDF downregulation in lLNv alone had no effect on activity rhythms under LD, showing that sLNv PDF is sufficient for M-activity [27]. However, whether sLNv PDF is necessary for M-anticipation is unclear, since, in the previous studies, a complete loss of PDF specifically from sLNv in both soma and DP was not achieved [26,27]. We established a phenotype of complete loss of PDF selectively from the sLNv (both in soma and DP) using a combination of genetic and environmental manipulations and tested the necessity of sLNv PDF for M-anticipation. We show that under LD, flies completely lacking PDF from sLNv exhibit M-peak with anticipation to lights-ON and correctly phased E-peak, showing unambiguously that sLNv PDF is not necessary for M-anticipation. PDF in lLNv is sufficient for this behaviour. Additionally, our finding that activity rhythms entrain to LD with a clear M-peak even when PER is undetectable in sLNv demonstrates that PER-driven clocks in sLNv are not necessary for M-peak.

## Materials and methods

### Fly lines

Transgenic fly lines with coding region for first 548 amino acids of *Htt* gene containing either non-pathogenic form without poly glutamine repeats (*w;+;UAS-HTT-Q0A*;+) or a pathogenic tract of 128 glutamine repeats (*w;UAS-HTT-Q128C*;+) were a gift from Troy Littleton, MIT [28]. In a previous study, the genetic background of flies was *yw* and even controls *yw/Q0*, *yw/Q128* and *pdfGal4/Q0* exhibit relatively poor rhythmicity—ranging about 60 to 70% [26]. To negate the possible influence of genetic background, we backcrossed the flies onto a *w*^*1118*^ background for over seven generations. Males of *UAS-HTT* were crossed with females of either *w*^*1118*^;*pdfGal4*;+ to obtain flies that expressed either non-pathogenic or pathogenic form of Huntingtin in PDF neurons (*pdf>Q0* or *pdf>Q128*) or with *w*^*1118*^;+;+ (BL 5905) to obtain UAS controls (*Q0* and *Q128*). *pdfGal* denotes the Gal4 control and *w*^*1118*^ served as the background control. For co-expression of HTT-Q and PDF in LNv neurons, *ywpdfGal4;UAS-PDF*:+ were crossed with UAS-HTT-Q lines to obtain *pdf>Q0*,*PDF* and *pdf>Q128*,*PDF*. *UAS-pdf* was a gift from Paul Hardin, Texas A&M University [7]. All fly lines and crosses were maintained on standard corn meal medium under 12:12h LD cycles at 25°C, unless specified otherwise. Subsequently, activity was recorded in specific light regimes from post-eclosion age day 3 (3d, henceforth age is denoted in this format) to capture changes from an early age.

### Behavioural assays

Virgin male flies (age 1-2d old) were housed individually in glass tubes (length-6.5cm, diameter-7mm) with one end having corn food (0.25 ml) and a seal of paraffin wax and the other end plugged with cotton. Their activity rhythms were recorded using the TriKinetics DAM system (TriKinetics, Waltham, MA). The assays were carried out at 25°C in incubators (Sanyo MIR-154 and Percival DR36VL). Raw time series data obtained during DD were analysed with the CLOCKLAB software (Actimetrics, Wilmette, IL) Chi Square periodogram with a cut-off of *p* = 0.01 [29]. To track progressive changes, the rhythm characteristics were quantified over three 7d age windows (AWs) comprising: AW1 (age 3d-9d early window), AW2 (age 10d-16d middle window) and AW3 (age 17d-23d late window). A fly was considered rhythmic if the periodogram amplitude was above the cut-off and this was confirmed with visual inspection of the actogram. The amplitude of the periodogram was used as a measure of rhythm robustness. To determine the extent of daily activity consolidation, 24h activity/rest time series data binned every 1h was converted into a polar co-ordinate system of a circle made of 24 points, such that the 24 equidistant points around the circle represented time of day and the corresponding activity at each of those 24 time-points. Each such point on the circle was resolved into their Cartesian *x* and *y* coordinates by respectively computing the product of the sine and cosine values of that time with activity counts in that hour, thus assigning to each time-point the amount of activity in the 1h bin. A vector sum of the resolved *x* and *y* coordinates normalised by daily total activity provided the X and Y vectors for daily activity distribution and the resultant of these two vectors is ‘*r’* (radius of the unit circle). This radial distance ‘*r*’ from the centre indicates the extent of activity consolidation. Due to the multiplication of sine and cosine values of each time-point with the activity at that time-point, a rhythmic fly having consolidated activity is expected to have a higher magnitude of ‘*r*’, whereas an arrhythmic fly with daily dispersed activity has a lower ‘*r*’ because of activity being multiplied to all the time-points in a day. Daily ‘*r*’ was averaged across flies. For LD, the mean activity profiles were plotted by averaging 15min activity counts over 7d for an individual and then averaging across flies. The measures for morning and evening anticipation, the anticipation index (AI) was quantified by a previously reported method as the ratio of activity counts 3h prior to dark/light or light/dark transition to the activity counts 6h prior to that transition [30]. Mean normalised activity profiles were plotted by averaging 15min binned individual fly raw activity over 5d, normalized by total activity of the individual and then averaged across flies.

#### Statistical analysis

For mean rhythmicity, period, rhythm robustness, ‘*r*’, activity levels and anticipation indices, a 2-way factorial ANOVA was carried out with genotype and age as the fixed factors. For between regime comparisons, a 2-way factorial ANOVA was carried out with regime and genotype or regime and age as the fixed factors. Post-hoc multiple comparisons in all the cases were carried out using Tukey’s Honest Significant Difference test at α = 0.05. For % rhythmicity and anticipation indices, the square root of data was Arcsine transformed and then used for statistical analyses. For number of rhythmic and arrhythmic flies, multiple pair-wise comparisons between genotypes or regimes were carried out using the 2x2 contingency table with a Yates Chi Square statistic. All statistical analyses were executed using STATISTICA^™^, version 7.

### Immunocytochemistry

A previously described immunocytochemical method was used [20]. Briefly, adult fly brains were dissected at specified ages in ice cold 1X PBS, fixed with 4% paraformaldehyde at room temperature (RT) for 30 minutes. 10% horse serum in 0.5% PBT was used as blocking-solution. For co-staining with anti-HTT, samples were incubated with blocking-solution for 1h at RT, 6h at 4°C and primary antibody for 48h at 4°C. For co-staining with anti-PER and anti-PDF, samples were incubated with blocking-solution for 1h at RT, overnight at 4°C and primary antibody for 48h at 4°C. For single staining with anti-PDF, the samples were incubated with blocking-solution for 1h at RT and with primary antibody for 24h at 4°C. Incubation with secondary antibodies was for 24h at 4°C. Post-immunostaining, whole brains were mounted on slides using 70% glycerol in 1X PBS.

Primary antibodies used were anti-Huntingtin Mouse (1:500) (Millipore MAB2166) along with anti-PDF Rabbit (1:30,000) (a gift from Michael Nitabach, Yale University), anti-PER Rabbit (1:20,000) (a gift from Jeffrey C Hall, Brandeis University) with anti-PDF Mouse (1:5000) (DSHB PDF C7). Secondary antibodies Alexa fluors (Invitrogen) (1:3000) anti-rabbit488, anti-rabbit546 and anti-mouse546 were used. To ascertain the status of PDF, *pdf>Q128* flies were grouped into rhythmic (Rhy) or arrhythmic (Arr) categories based on the method described above and were sampled on alternate days for dissection at CT2-4 following from the previous LD. Since most *pdf>Q128* flies are arrhythmic, a small number of rhythmic flies were available for dissection at age 7d and age 15d. For PDF oscillations in sLNv DP, *pdf>Q128* and *Q128* flies were dissected at age 9d at CT2-3 (CT2), CT6-7 (CT6), CT10-11 (CT10), CT14-15 (CT14), CT18-19 (CT18) and CT22-23 (CT22) in DD (7d in DD) or ZT2-3 (ZT2) and ZT11-12 (ZT11) in LD and stained with anti-PDF rabbit (1:30,000). For PER oscillations, *pdf>Q128* and *pdf>Q0* were dissected at CT23-24 (CT23), CT5-6 (CT5), CT11-12 (CT11), CT17-18 (CT17) or at ZT23-24 (ZT23), ZT5-6 (ZT5), ZT11-12 (ZT11), ZT17-18 (ZT17). These samples were co-stained with anti-PDF to enable identification of LNv. We considered a change in PER intensity across time-points as a circadian oscillation when intensity at a time-point is statistically different from its neighbouring time-points on either side. For detecting PDF in sLNv DP in flies reared in constant light (LL), a higher concentration of anti-PDF rabbit (1:10,000) was used so as enable detection of very low levels of PDF. In most flies expressing pathogenic Huntingtin in LNv, we are unable to detect PDF in sLNv soma; although we cannot rule out its presence at extremely low levels. However, even on increasing antibody concentration to thrice the usual levels, we are unable to detect PDF in sLNv soma (S5 Fig), suggesting that PDF in sLNv soma is possibly negligibly small or altogether eliminated. In contrast to PDF in sLNv soma, PDF is easily detected in sLNv DP and lLNv. A higher concentration of anti-PER Rabbit (1:5000) was used to enable detection of low levels of PER in *pdf>Q128*. All other details can be found in S1 methods.

## Results

### Pathogenic Huntingtin expressing flies show a selective loss of PDF from sLNv soma, while being present in the dorsal projections

**Fig 1:**
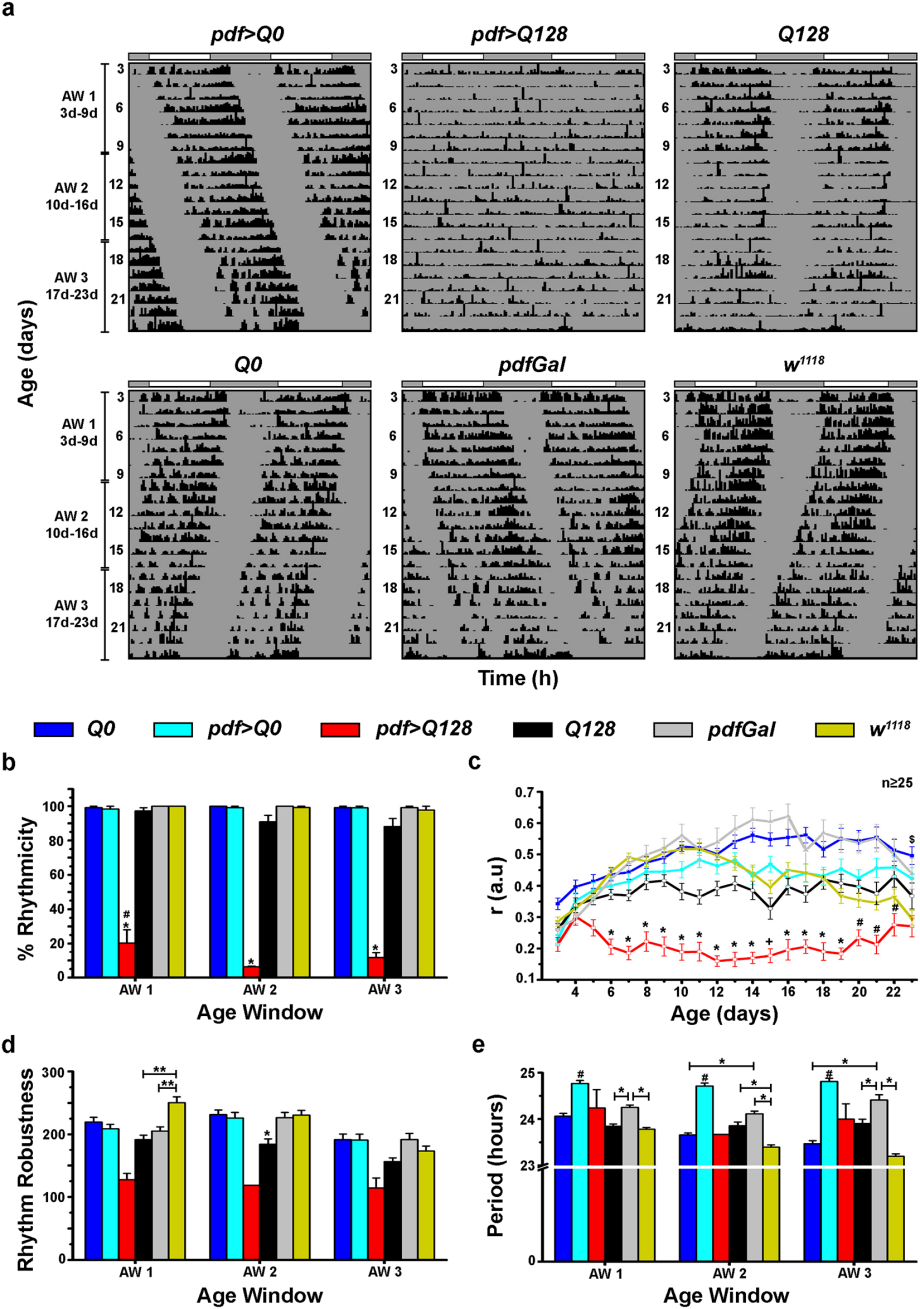
Flies that express pathogenic Huntingtin in LNv are arrhythmic in constant darkness. (a) Representative double plotted, normalized actograms (*x*-axis = 48h) for *pdf>Q0*, *pdf>Q128*, *Q128*, *Q0*, *pdfGal* and *w*^*1118*^ depicting activity data for >21days in DD (age 3d-23d on y-axis). The 21d data has been divided into three 7d age windows (AWs) namely AW1 (3d-9d), AW2 (10d-16d) and AW3 (17d-23d) as depicted on the left side. The white and grey bars above actograms represent light and dark phases of the previous LD. (b) The percentage of rhythmic flies averaged over four independent experiments is plotted across three AWs. *pdf>Q128* has significantly poor rhythmicity compared to other genotypes (* *p*<0.001). Significantly higher fraction of *pdf>Q128* flies are rhythmic in AW1 compared to AW2 (# *p*<0.05). (c) Mean ‘*r’* per day across age. Symbols indicate statistically significant differences at *p*<0.05: * *pdf>Q128* from other genotypes, ^+^ *pdf>Q128* from other genotypes except *Q128*, # *pdf>Q128* from other genotypes except *Q128* and *w*^*1118*^ and $ *pdf>Q128* from *pdf>Q0* and *Q0*. n≥25 indicates that for all genotypes, at least 25 flies remained alive at age 23d. (d) Mean robustness of rhythmic flies across age. In AW1, *w*^*1118*^ has significantly more robust rhythms than *Q128* and *pdfGal* (** *p*<0.01). In AW2, *Q128* has relatively less robust rhythms than other controls (* *p*<0.05). *pdf>Q128* have not been considered for statistical tests as very few of them were rhythmic. (e) Mean free running period across AWs. Symbols indicate statistically significant differences. *pdf>Q0* has a longer period than other genotypes (# *p*<0.01) across AWs. * indicates *p*<0.01. *pdf>Q128* have not been considered for statistical tests as very few of them were rhythmic. Across all panels, error bars are SEM.

A previous study showed that expression of pathogenic Huntingtin (HTT-Q128) in PDF expressing LNv (henceforth denoted as PDF^+^ LNv) results in flies showing arrhythmic locomotor activity in constant darkness as early as 3d, with a selective loss of PDF only from the sLNv [25,26]. But, in that study the rhythmicity of control flies was low [26] and therefore, we backcrossed the flies to reduce background effects. We see that *pdf>Q128* flies are arrhythmic from the first day in DD (Top-middle panel in Fig 1a), whereas all controls are rhythmic (Fig 1a) and the percentage of rhythmic *pdf>Q128* flies is significantly lower than controls across AWs (Fig 1b). Thus, in a *w*^*1118*^ background, despite the controls exhibiting close to 90–100% rhythmicity across age, HTT-Q128 expressing flies are mostly arrhythmic in DD, concordant with the previous study [26]. To obtain a greater temporal resolution of change in rhythm features (daily, as opposed to 7d AWs), we estimated ‘*r*’, an indicator of the consolidation of daily activity. We find that *pdf>Q128* flies show poor daily activity consolidation ‘*r*’ than controls for a major part of the recording, beginning at age 6d, up to 19d, (at which time, controls also begin to show a reduction in ‘*r’*) (Fig 1c). Flies expressing the non-pathogenic form HTT-Q0 (*pdf>Q0*) show robust rhythms comparable to *pdfGal* and *w*^*1118*^ (Fig 1d) with a consistently long period, which is partly reflective of the long period of the genetic background of its parent, the driver *pdfGal4* (Fig 1e). A small fraction of *pdf>Q128* flies are weakly rhythmic evidenced by very low values for robustness and exhibit close to 24h period (Fig 1d and 1e).

**Fig 2:**
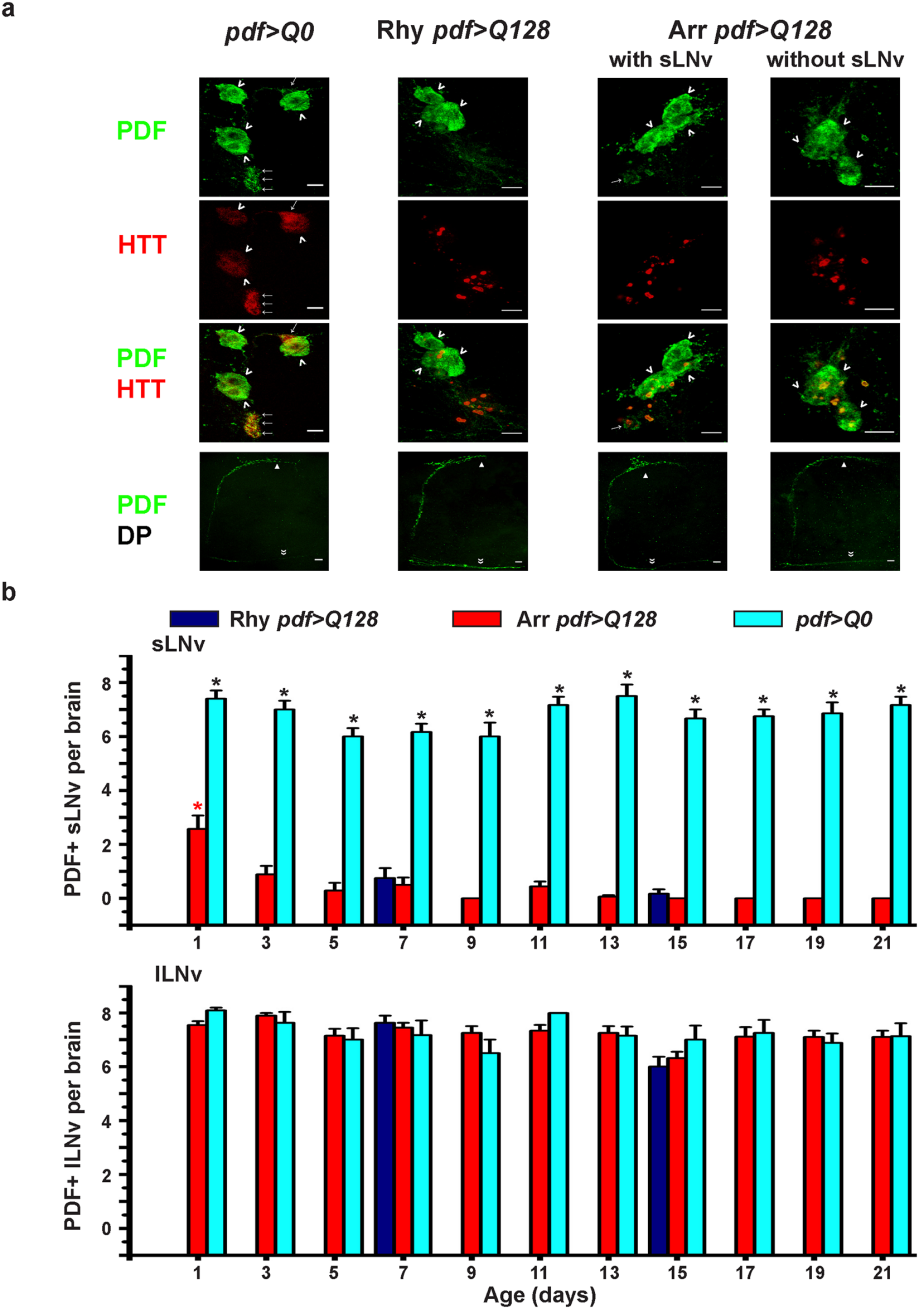
Weakly rhythmic and arrhythmic *pdf>Q128* flies mostly lack PDF^+^ sLNv soma, while it is present in their DP. (a) Representative images of adult brains of rhythmic *pdf>Q0* (age 11d), rhythmic *pdf>Q128* (age 7d) and arrhythmic *pdf>Q128* (age 11d) stained for PDF (green) and HTT (red) showing sLNv soma (arrows), lLNv soma (arrowheads), sLNv dorsal projections (triangles) and lLNv contralateral projections (double arrowheads). Examples of arrhythmic *pdf>Q128* with one sLNv soma (third column) and no sLNv (fourth column) are shown. Scale bars are 10 μm. (b) Top: Average number of PDF^+^ sLNv soma per brain for rhythmic *pdf>Q0* flies and arrhythmic *pdf>Q128* flies across age and for rhythmic *pdf>Q128* at ages 9d and 15d. Symbols indicate statistically significant differences: * (black) between *pdf>Q128* and *pdf>Q0* for each age (*p*<0.0001), * (red) between *pdf>Q128* at age 1d from both rhythmic and arrhythmic *pdf>Q128* at other ages (*p*<0.001). Bottom: Average number of PDF^+^ lLNv soma per brain plotted as in top panel. n = 6–10 whole brains/genotype/age. Across all panels, error bars are SEM.

We asked whether the weakly rhythmic *pdf>Q128* flies have more number of PDF^+^ sLNv compared to their arrhythmic counterparts. A previous report suggests that the presence of a single LNv is sufficient to elicit rhythmicity in DD if the LNv terminals reach the superior protocerebrum [31]. Across age, *pdf>Q128* flies (Rhy and Arr) have significantly fewer PDF^+^ sLNv soma (Fig 2a and 2b) and its frequency distribution across age shows a left skew towards 0 PDF^+^ sLNv soma that is significantly different from the right skewed distribution of *pdf>Q0* (Panel a in S1 Fig). In contrast, the PDF^+^ lLNv soma numbers and distributions are comparable between genotypes (Fig 2a and 2b, Panel a in S1 Fig). Consistent with reported results [25,26], most *pdf>Q128* flies showed loss of PDF from sLNv soma (Fig 2a). Interestingly, in *pdf>Q128* flies, the number (Fig 2b, top blue *vs*. red bars) and frequency distribution (Panel a in S1 Fig) of PDF^+^ sLNv soma between rhythmic and arrhythmic individuals is not different at ages 7d and 15d. Importantly, both rhythmic and arrhythmic *pdf>Q128* flies have PDF in sLNv DP (Fig 2a). Pooling weakly rhythmic flies of ages 7d and 15d, about 71% of flies (10/14 brain samples) do not have detectable PDF in sLNv soma but have PDF in DP (Middle panel in Fig 2a) suggesting that their residual rhythmicity might stem from PDF in DP or non-sLNv mechanisms. In a very small proportion of arrhythmic flies (10/73) at least one PDF^+^ sLNv soma is detected up till age 15d (Third column in Fig 2a and Panel a in S1 Fig); however, regardless of PDF absence in sLNv soma, these arrhythmic flies exhibit PDF in sLNv DP (Third and fourth columns in Fig 2a). This reveals that presence of PDF^+^ sLNv with intact DP does not ensure rhythmicity. In summary, we show that for most *pdf>Q128* flies weakening or breakdown of activity rhythms is associated with loss of PDF from sLNv soma, while being present in sLNv DP and lLNv.

Since the total number of *pdf>Q128* flies with PDF in sLNv soma were small, we did not quantify possible differences in levels or cellular distribution of PDF in these cells. In a separate experiment, at the middle of AW1 (age 5d-6d), where a sufficiently high number of flies are expected to have at least one PDF^+^ sLNv, we find no difference between PDF levels in soma of sLNv or lLNv between *pdf>Q128* and *pdf>Q0* (Panel b in S1 Fig). In an attempt to overcome effects of HTT-Q128 by overexpression of PDF in LNv, we co-expressed HTT-Q128 and PDF in LNv (*pdf>Q128*,*PDF*); however, such flies are arrhythmic like *pdf>Q128*, and the percentage rhythmicity is significantly lower than controls (Panels c and d in S1 Fig).

### PDF oscillations in sLNv dorsal projections and synchronous PER oscillations in PDF^-^ circadian neurons persist in the absence of PER in sLNv

**Fig 3:**
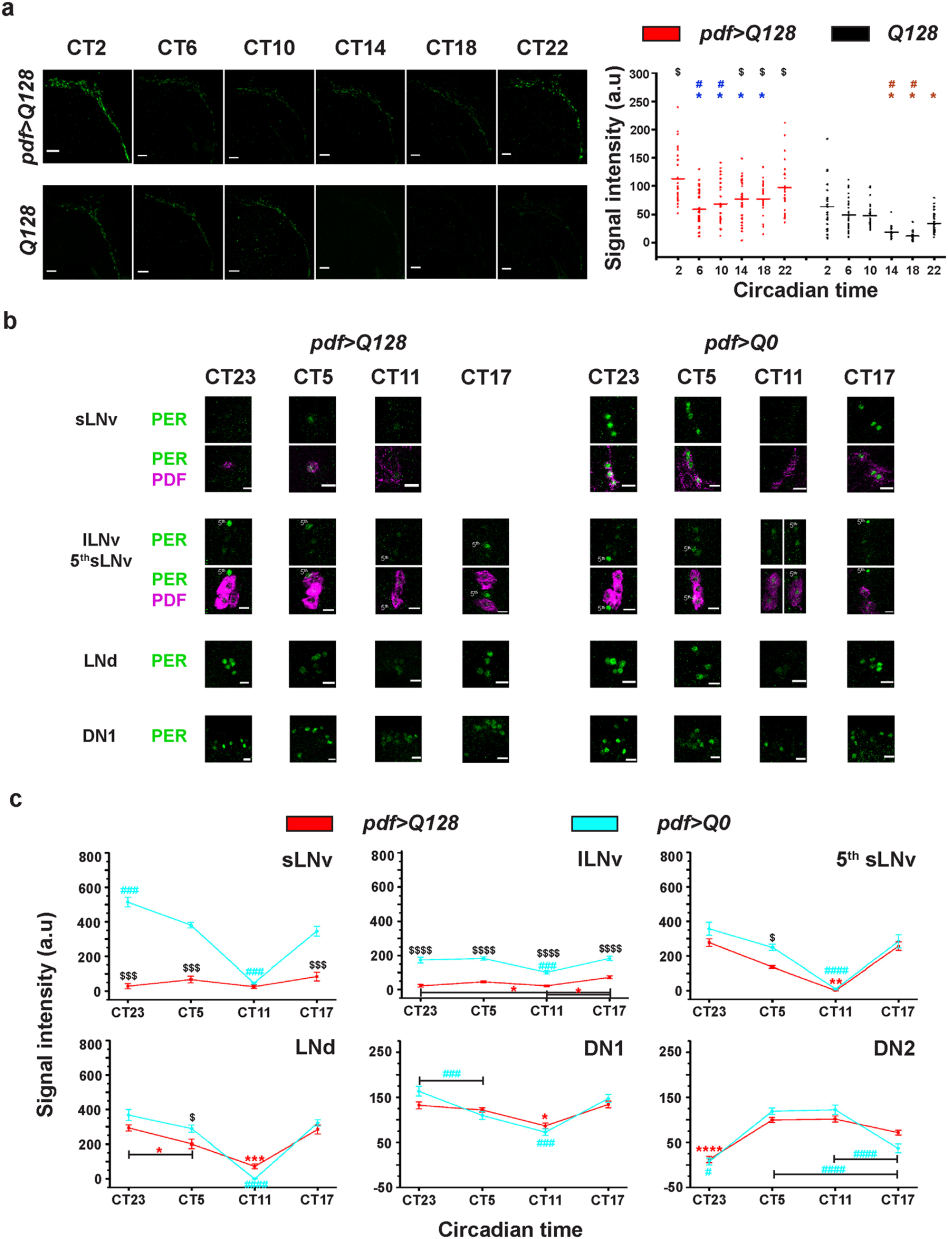
Oscillations of PDF in sLNv dorsal termini and synchronous oscillations of PER in other circadian neurons persist despite loss of PDF in sLNv soma and loss of PER in LNv. (a) Left: Representative images of 9d old adult brains showing PDF (green) in sLNv dorsal projections (DP) at six time-points in DD for *pdf>Q128* and *Q128*. Scale bars are 10 μm. Right: Quantification of PDF intensity in sLNv DP, across time-points, for both genotypes with each circle representing individual hemisphere values and horizontal line depicting the mean value. Symbols indicate statistically significant difference: * (blue) of CT2 from CT6-CT18 for *pdf>Q128* at *p*<0.01, # (blue) of CT22 from CT6-CT10 for *pdf>Q128* at *p*<0.05, * (brown) of CT2 from CT14-CT22 for *Q128* at *p*<0.05, # (brown) of CT6-CT10 from CT14-CT18 for *Q128* at *p*<0.05 and $ (black) between *pdf>Q128* and *Q128* at indicated time-points at *p*<0.0001. Error bars are SEM. n≥26 hemispheres/genotype/time-point. (b) Representative images of 9d old adult brains stained for PER (green) in different circadian neuronal groups at four time-points in DD for *pdf>Q128* and *pdf>Q0*. LNv soma is identified by its co-staining with PDF (magenta). At CT17, there is no representative image showing sLNv in *pdf>Q128*. Scale bars are 10 μm. (c) Quantification of PER intensity across time-points in various circadian neuronal groups for *pdf>Q128* and *pdf>Q0* in DD. Very few *pdf>Q128* flies had detectable PDF^+^ and PER^+^ sLNv soma. Symbols indicate statistically significant difference: * (red) between time-points for *pdf>Q128*, # (blue) between time-points for *pdf>Q0* and $ between genotypes within a time-point with single symbol *p*<0.05, double symbols *p*<0.01, triple symbols *p*<0.001 and quadruple symbols *p*<0.0001. n = 16–20 hemispheres/genotype/time-point. Across all panels, error bars are SEM.

Flies expressing pathogenic Huntingtin are behaviourally arrhythmic in DD and PDF is not detectable in soma of sLNv although detectable in their DP (Fig 2). Previous studies using different approaches have suggested that oscillations in PDF levels in sLNv DP is crucial for synchronising the molecular oscillations between various circadian neuronal groups in DD [14,16,18,21]. To assess whether PDF detected in sLNv DP of *pdf>Q128* flies oscillates, we examined brains of 9d old adult flies reared under LD and transferred into DD at age 2d. *pdf>Q128* shows oscillation in PDF levels with intensity at CT2 significantly higher than CT6-CT18 (Fig 3a). Control *Q128* shows oscillation in PDF levels with intensity at CT2 significantly higher than CT14-CT22 (Fig 3a). The PDF peak at CT2 of *pdf>Q128* and *Q128* are in-phase. However, the rise and fall of PDF levels in *pdf>Q128* is earlier than *Q128* (Fig 3a) and is suggestive of a short period PDF oscillation in *pdf>Q128*. Across most time-points, PDF levels in *pdf>Q128* are higher than *Q128*, but the peak to trough differences are qualitatively comparable. Oscillating PDF levels in sLNv DP are not direct measurements of rhythmic secretion; but we reasoned that the functional consequence of such secretion would be to synchronise molecular oscillations among circadian neurons. Hence, we assessed oscillations of PER levels in different circadian neuronal subsets. In *pdf>Q128*, PER is mostly not detected in sLNv soma (Fig 3b), and a frequency distribution of PER^+^ sLNv soma shows that most hemispheres have no PER^+^ sLNv soma which is significantly different from distribution of *pdf>Q0* (Panel a in S2 Fig). Pooling across time-points, in 83.3% of hemispheres neither PER nor PDF is detected in sLNv soma, in 7.7% both PER and PDF are detected in sLNv soma, in 5.9% only PDF is detected and in 3.1% only PER is detected. The number and distribution of PER^+^ sLNv soma resembles that of PDF^+^ sLNv soma (Panel a in S2 Fig). In controls, PER in sLNv oscillates with a trough at CT11 and a peak at CT23 (Fig 3b and 3c). The very few PER^+^ sLNv detected in *pdf>Q128* have intensity lower than control at CT23, CT5 and CT17 (Fig 3b and 3c). Even on increasing antibody concentrations four-fold, we are mostly unable to detect PER^+^ sLNv in *pdf>Q128* (Panel b in S2 Fig). Thus, pathogenic HTT expression in LNv results in loss of detectable PER from sLNv soma. Among PDF^+^ lLNv soma of *pdf>Q128*, only about half are PER^+^ (Panel c in S2 Fig). In *pdf>Q0*, lLNv soma PER shows dampened oscillations of very low amplitude (Fig 3b and 3c). PER in lLNv soma of *pdf>Q128* does not oscillate and has significantly poor intensity compared to *pdf>Q0* across time-points (Fig 3b and 3c). Thus, in *pdf>Q128* flies even though PDF levels in lLNv soma and PDF^+^ lLNv numbers are comparable to controls (S1 Fig and Fig 2), PER expression in lLNv soma is lowered. 5^th^ sLNv of *pdf>Q128*, like its control, shows a trough in PER oscillations at CT11 (Fig 3b and 3c) and its amplitude is mostly comparable and in-phase with its controls. In LNd of *pdf>Q128*, like controls, PER shows a clear oscillation with a trough at CT11, (Fig 3b and 3c) and its levels are mostly similar to *pdf>Q0*. In DN1s of *pdf>Q0* and *pdf>Q128*, there is a clear PER oscillation of similar amplitude with a trough at CT11 (Fig 3b and 3c). *pdf>Q128* and *pdf>Q0* show PER oscillations in DN2 with a trough at CT23 (Fig 3c). Interestingly, the trough of PER intensity within each neuronal group in *pdf>Q128* is phased similar to their counterparts in *pdf>Q0* at CT11 (Fig 3c). Also, in *pdf>Q128*, in the absence of PDF in sLNv soma, PER intensity oscillations in 5^th^ sLNv, LNd and DN1 (henceforth referred to as PDF^-^ neurons) are in-phase. PER intensity in DN2 shows a trough at CT23 in both genotypes and is phase advanced compared to other neuronal groups, which is in accordance with a previous study [32]. Further, as a measure of within-group synchrony, for LNd and DN1 neuronal groups we estimated the standard deviation of PER intensity per hemisphere. We find that this measure for *pdf>Q128* is not higher than controls for most time-points (S1 Table), suggesting that within-group synchrony is not affected. We interpret this synchrony in PER intensity oscillations between PDF^-^ neurons as indirect evidence that rhythmic PDF accumulation in sLNv DP of *pdf>Q128* flies is functional and perhaps indicative of rhythmic PDF secretion. In summary, we find that expression of pathogenic Huntingtin in LNv results in reduction of PER levels to below detection limit in sLNv and PER being a central clock component, we interpret this as a disruption of the free-running molecular clock in sLNv. In lLNv, we find that PER levels are reduced; however, since PER oscillations in lLNv dampen in DD even in wild-type flies and are shown to be not essential for circadian activity rhythms in DD [4,14,33], we associate breakdown of rhythmic activity with sLNv circadian dysfunction. The persistence of PDF oscillations in sLNv DP and synchronous molecular oscillations in PDF^-^ neurons in these arrhythmic flies suggest that these oscillations are not sufficient for activity rhythms in DD. Continuation of oscillating PDF levels in sLNv DP in the absence of detectable PER in the sLNv soma suggests that these oscillations are not dependent on PER-driven molecular clocks in the soma. Overall, we find that the circadian pacemaker function of sLNv for behavioural rhythmicity in DD is compromised in *pdf>Q128* flies, in a manner independent of PDF oscillations in sLNv DP and subsequent synchrony in molecular oscillations between other circadian neurons. Thus, these results challenge the existing notion that the sLNv evoke self-sustained activity rhythms in DD via oscillations of PDF in its DP.

### Circadian molecular clock in sLNv is not necessary for entrainment to LD

**Fig 4:**
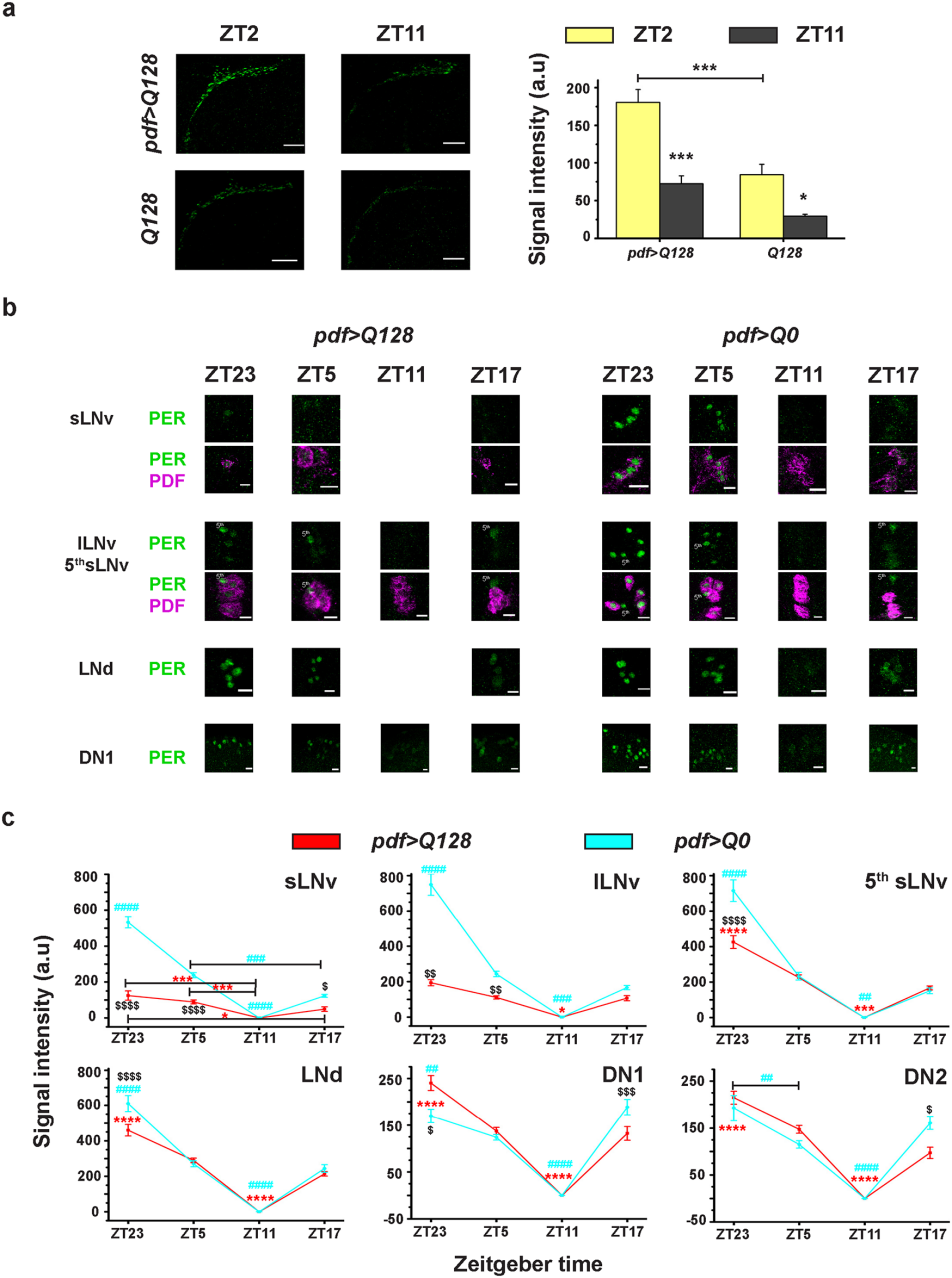
Under LD PER is lost from sLNv and PER intensity oscillations in lLNv are dampened in flies expressing pathogenic Huntingtin. (a) Left: Representative images of adult brains showing PDF (green) in sLNv DP at two time-points under LD for *pdf>Q128* and *Q128*. Scale bars are 20 μm. Right: Quantification of PDF intensity in sLNv DP across time-points where both genotypes show a diurnal oscillation. * *p*<0.05, *** *p*<0.0001. n = 18–24 hemispheres/genotype/time-point. (b) Representative images of adult brains stained for PER (green) in different circadian neuronal groups at four time-points in LD for *pdf>Q128* and *pdf>Q0*. PDF (magenta) is co-stained to identify LNv soma. At ZT11, in most samples PER levels was very low and not visible. There are no representative images showing PER in sLNv and LNd of *pdf>Q128* at ZT11. Scale bars are 10 μm. (c) Quantification of PER intensity across time-points in various circadian neuronal groups for *pdf>Q128* and *pdf>Q0* under LD. All other details are same as Fig 3c. Across all panels, error bars are SEM.

PDF has been shown to be critical for anticipation of dark-light transition (M-anticipation) [7]. A previous study showed that pathogenic Huntingtin expressing flies lacking PDF in sLNv soma exhibits M-anticipation under high intensity LD (1000–2500 lux) [26] and we demonstrate that these flies continue to show M-anticipation even under low intensity LD (100 lux) (S3 Fig). Moreover, *pdf>Q128* like its control *Q128* shows a clear oscillation in PDF levels in sLNv DP: intensity at ZT2 is significantly higher than ZT11 (Fig 4a). We also examined whether the circadian molecular oscillations are sustained in sLNv of *pdf>Q128* flies in the presence of LD cycles. On average, fewer PER^+^ sLNv soma are found in *pdf>Q128* compared to *pdf>Q0* (Fig 4b and Panel a in S4 Fig) and their mean numbers and distribution closely corresponded to the number of PDF^+^ sLNv soma (Panel a in S4 Fig). The few PER^+^ sLNv soma detected in *pdf>Q128*, have significantly poorer intensity at ZT23 and ZT5 than *pdf>Q0* which shows clear oscillations of PER in sLNv (Fig 4b and 4c). Thus, even in the presence of cyclic light cues, PER is largely undetected in sLNv soma, suggesting that the molecular clock in sLNv is indeed impaired. PER and PDF in lLNv soma of *pdf>Q128* are comparable in number and distribution to lLNv PER and PDF of *pdf*>Q0 (Fig 4b and Panel b in S4 Fig). In these neurons, PER shows a clear oscillation with a trough at ZT11 under LD (Fig 4b and 4c); although they are of low amplitude compared to *pdf>Q0*, which show robust PER oscillations (Fig 4b and 4c). In *pdf>Q128*, even in the absence of PDF and PER in sLNv soma, oscillations in PER levels are in-phase across neuronal groups and with controls, with a trough at ZT11 (Fig 4b and 4c). Thus, under LD, despite loss of PDF and PER from sLNv soma, there is rhythmic PDF accumulation in sLNv DP and persistence of M-anticipation. The photic entrainment of activity rhythms of flies lacking PER oscillations in sLNv suggests that sLNv molecular clock is not essential for this phenomenon.

### PDF in sLNv is not necessary for morning anticipation under light/dark cycles

**Fig 5:**
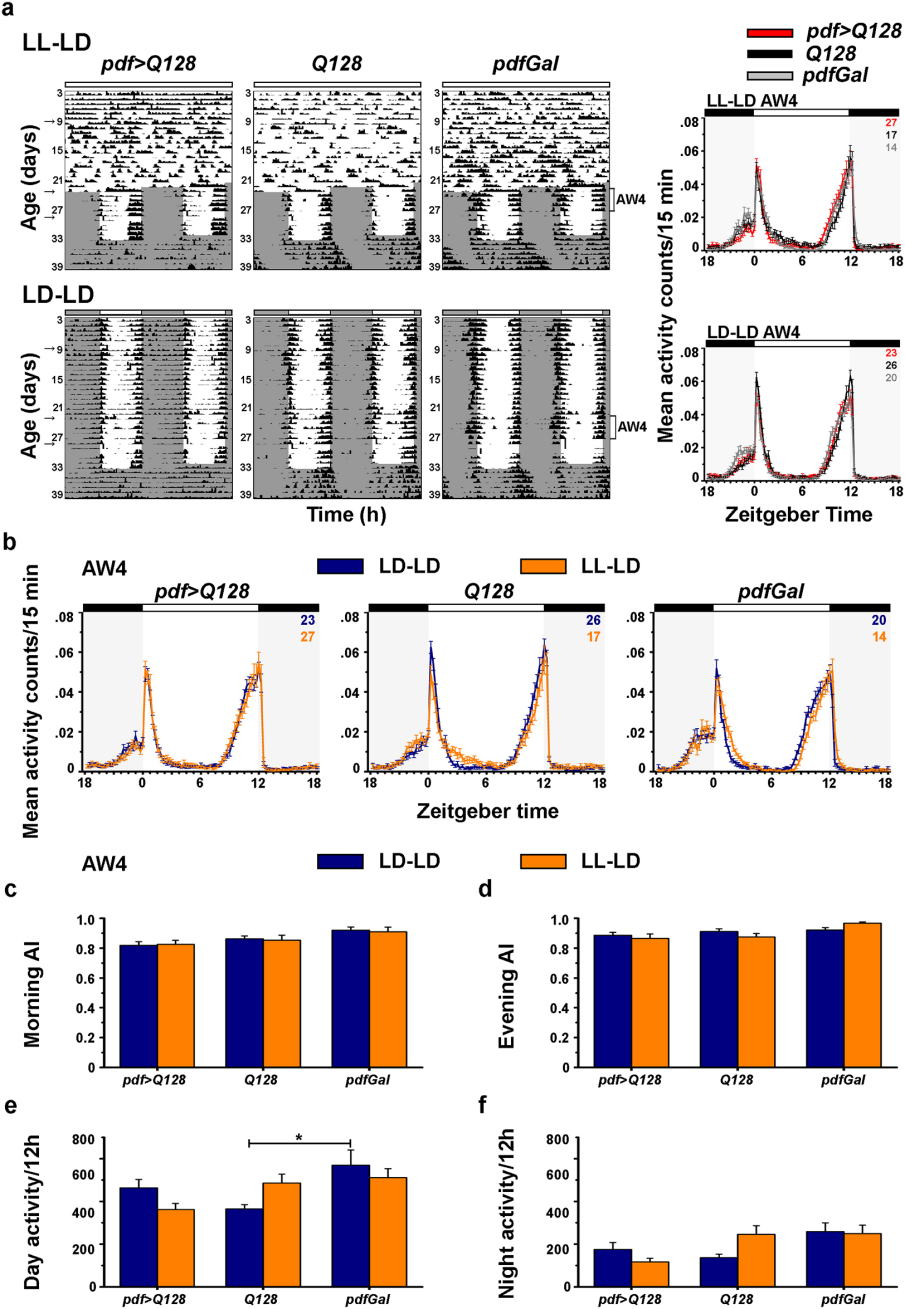
Flies that lack PDF in sLNv exhibit morning and evening anticipation. (a) Left: Representative double plotted, normalised actograms for *pdf>Q128*, *Q128* and *pdfGal* in LL-LD (top) and LD-LD (bottom). The horizontal unshaded bar on top in LL-LD depicts developmental light regime LL. The grey shaded regions represent dark phase and unshaded regions represent light phase of LD. All other details are similar to Fig 1a. In LL-LD and LD-LD, AW4 (age 24d-28d) is indicated. Gap in the actograms is due to interruption of recording. Arrows at ages 9d, 23d and 28d indicate the ages at which dissections were carried out. Right: Mean normalized activity counts per 15 min comparing *pdf>Q128*, *Q128* and *pdfGal* in LL-AW4 (top) and under LD-AW4 (bottom). The grey shaded regions and black horizontal bars above represent dark phase and the unshaded region and white horizontal bars above represent light phase of the LD. The coloured numbers at top right of each panel is the sample size for each genotype. (b) Mean normalized activity counts per 15 min comparing LL-LD with LD-LD for each genotype in AW4. All other details are similar to Fig 5a right. (c-f) Across genotypes in AW4, there are no significant differences between regimes LL-LD and LD-LD in terms of morning anticipation index, evening anticipation index, mean daytime activity counts per 12h and mean night-time activity counts per 12h. * indicates significant difference at *p*<0.01. Across all panels, error bars are SEM.

The ability of *pdf>Q128* flies to exhibit M-anticipation in the absence of PDF in sLNv soma could be due to oscillating PDF in the sLNv dorsal projections and conveying time information to the rest of the circuit, or a PDF-independent light-dependent process. Ongoing studies in the laboratory had revealed that *pdf>Q128* flies raised and maintained in constant light (LL) show a progressive loss of PDF from sLNv DP over time with near complete loss by 23d, whereas their age-matched *pdf>Q0* controls remain unaffected (Panel b in S5 Fig). Therefore, we used this regime to generate flies lacking PDF in sLNv termini to determine the necessity of PDF cycling in projections for M-anticipation under LD. One group of flies were maintained in LL (about 200 lux) during development and activity recorded as adults in LL up to 23d, followed by LD for 10d followed by DD (denoted by superscript LL-LD, top left in Fig 5a). In the control regime, flies were reared and recorded under LD up to 33d followed by DD (denoted by superscript LD-LD, bottom left in Fig 5a). In *pdf*>Q128^LL-LD^ flies, no PDF^+^ sLNv soma are detected at age 23d and 28d, similar to *pdf*>Q128^LD-LD^ (Panels a and c in S5 Fig), and PDF^+^ lLNv soma numbers are comparable between regimes (Panels a and c in S5 Fig). *pdf>Q128*^LL-LD^ flies show presence of PDF in sLNv DP in all samples at age 9d, but by age 23d, PDF in sLNv DP is lost in about 80% of the hemispheres, while present in all *pdf>Q128*^LD-LD^ flies up to 28d (Panels a and c in S5 Fig). PDF is present in lLNv contralateral projections across age in both regimes (Panels a and c in S5 Fig). In *pdf>Q128*^LL-LD^ at age 28d, PDF is undetectable in sLNv DP even after experiencing 5d of LD (Panel c in S5 Fig). To address the question of necessity of PDF in sLNv DP for M-anticipation, we focussed our behavioural analysis to this 5d age window of 24d-28d (AW4). In AW4, both *pdf>Q128*^LL-LD^ and *pdf>Q128*^LD-LD^ flies exhibit activity profiles similar to their respective within-regime controls (Right panels in Fig 5a). The profile of *pdf>Q128*^LL-LD^ (where PDF is absent in sLNv DP) is also similar to that of *pdf>Q128*^LD-LD^ (where PDF is present in sLNv DP) with similarly phased morning and evening peaks and gradual build-up of activity before the dark/light and light/dark transitions (Left panel in Fig 5b). Morning and evening AIs of *pdf>Q128*^LL-LD^ are not different from *pdf>Q128*^LD-LD^ or from *Q128* and *pdfGal* in LL-LD regime (Fig 5c and 5d) and their daytime and night-time activity levels are also similar (Fig 5e and 5f). Thus, M-anticipation does not require oscillating PDF in sLNv DP, since in the absence of PDF in sLNv DP (*pdf>Q128*^LL-LD^), flies entrain to LD similar to controls (Fig 5b–5d). As *pdf>Q128*^LL-LD^ flies in LL-LD lack PDF in sLNv (soma and DP), our results demonstrate that PDF in sLNv is dispensable for M-anticipation, so long as PDF is present in lLNv.

## Discussion

### Role of sLNv for activity rhythms in DD

We find that a majority of both weakly rhythmic and arrhythmic *pdf>Q128* flies show loss of PDF from sLNv soma, while present in sLNv DP and lLNv, suggesting that for the most part PDF in sLNv soma is critical for robust rhythms. Our results show that presence of non-zero sLNv (at least one) with control levels of PDF and intact DP is not always associated with behavioural rhythmicity. This is in contrast to *disco* mutants, where presence of single LNv with intact DP was sufficient for a fly to be rhythmic [31]. Unlike *disco* mutants where the sLNv when present with DP, are functional like wildtype, on expression of pathogenic Huntingtin, despite PDF presence in sLNv, other functional components of sLNv are likely to be compromised. This suggests that cellular features of PDF distribution, overall health and functionality of the sLNv rather than mere PDF presence is important in determining rhythmicity.

Expression of pathogenic Huntingtin in LNv seems to specifically affect sLNv soma where PDF and PER are lost, whereas sLNv DP seems to be unaffected in terms of PDF oscillations. Behavioural arrhythmicity in DD reflects sLNv dysfunction. Our results of behavioural arrhythmicity in DD, despite PDF oscillation in sLNv projections, brings to question the notion that control of rhythmic locomotor activity by sLNv is purely driven by oscillating PDF in its DP. Prior studies with genetic manipulations affecting sLNv functions result in loss of behavioural rhythmicity in DD and an associated loss or altered phasing of PDF oscillations in their DP [7,16–19,24,34]. In many cases, only the PDF oscillations are affected with the sLNv molecular clock being intact [16–18], suggesting that PDF is a critical sLNv output. Thus, in DD, rhythmic PDF accumulation and perhaps secretion is thought to be the functional outcome of sLNv leading to rhythmic behaviour. The short period rhythms seen in *pdf*^*01*^, hyperactivated LNv in the absence of PDF or LNv silenced flies is hypothesised to be a reflection of the short period clocks of LNd and DN in the absence of resetting signal in the form of rhythmic PDF [14,18,20].

**Fig 6:**
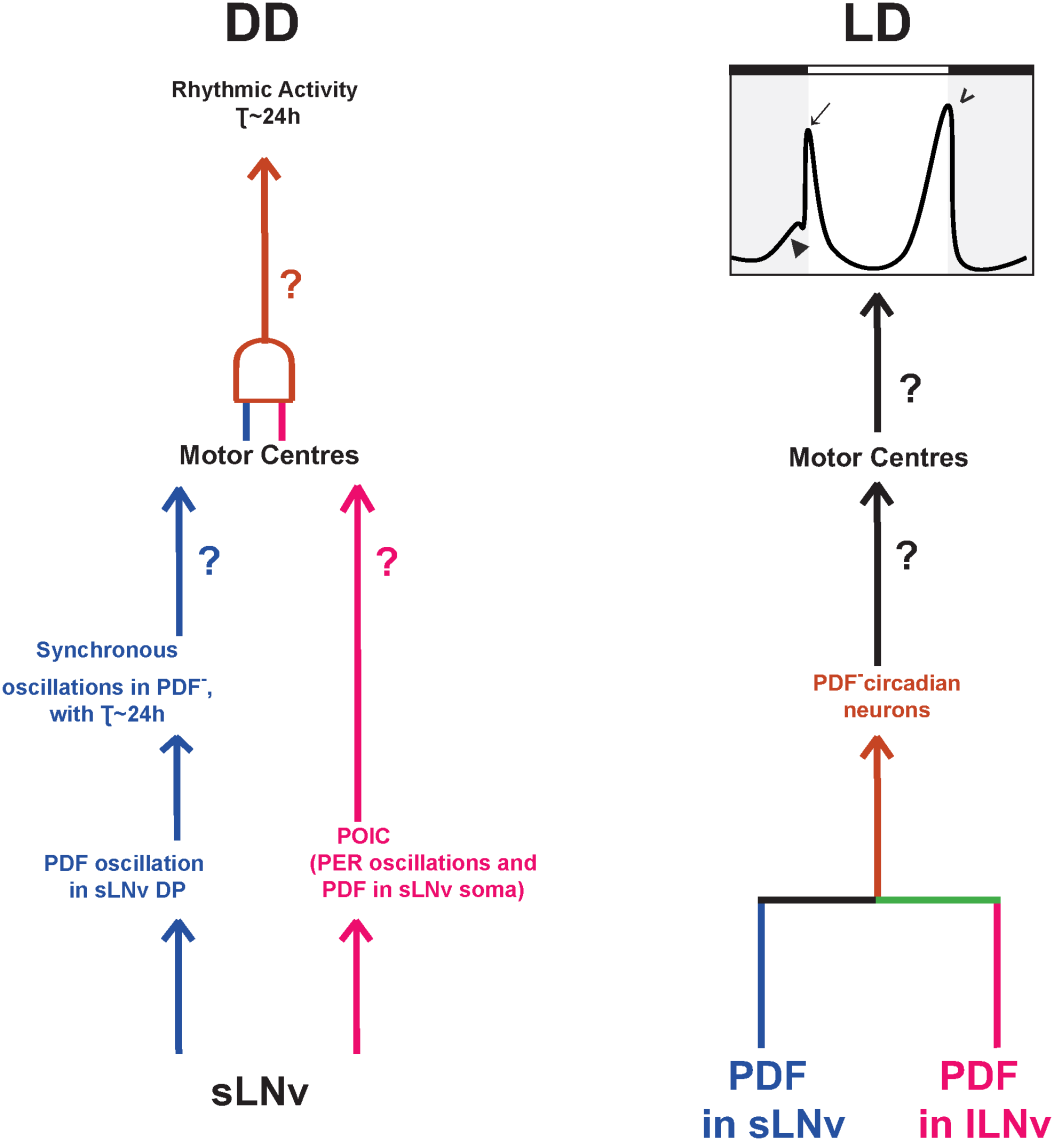
Model for how LNv sustains rhythmic activity in DD and modulates the temporal profile of the rhythms under LD. Left: (blue) Previous studies have suggested that sLNv sustains rhythmic activity in DD via rhythmic accumulation and likely secretion of PDF from its DP, synchronising the molecular clocks between PDF^-^ circadian neuronal groups and setting their period to near 24h [14,16–18] and the latter possibly communicating time information to motor centres (MC) [37]. We propose an additional component (magenta) for control of rhythmicity by sLNv, the POIC (PDF Oscillation Independent Component) that possibly involves PDF in sLNv soma and sLNv molecular clock. We propose that both inputs are necessary (brown) for near 24h activity rhythms in DD. Right: Previous studies have shown that, under LD, LNv PDF is critical for M-peak (arrow), M-anticipation (triangle) and E-peak phasing (arrowhead) and that sLNv PDF is sufficient [27] (blue). We show that for this behaviour lLNv PDF is also sufficient (magenta). Communication from LNv to PDF^-^ neurons via PDFR can bring about this behaviour [11] (brown) and a recent study provides a means for direct synaptic communication between lLNv with LNd [38] (green). We, therefore propose that PDF inputs from either sLNv or lLNv to PDF^-^ neurons are sufficient and the latter communicate to MC via yet unknown mechanisms to shape the activity profile under LD.

We show via immunocytological measurements that PDF levels oscillate in sLNv DP in DD in behaviourally arrhythmic flies. Although this is not direct evidence for rhythmic PDF secretion, based on previous studies which have shown that absence of PDF [14,21] or PDFR [9,35] results in loss of synchrony among the rest of the circadian neuronal circuit, we interpret our finding of synchronous PER oscillations in PDF^-^ clock neurons as indirect evidence for functionality of oscillating PDF levels in the DP. Direct measurements of PDF release as demonstrated at the larval neuromuscular junction [36] may be able to confirm this phenomenon. Additionally, the few flies that were weakly rhythmic had period close to 24h, indicating that oscillating PDF in sLNv DP functions by synchronising the PDF^-^ neuronal oscillators to run with near 24 periods. However, the PDF oscillation dependent resetting does not seem to be sufficient for behavioural rhythmicity, since, in the face of rhythmically accumulating PDF in sLNv DP and likely rhythmic secretion that enables synchrony of molecular oscillations in PDF^-^ neurons, locomotor activity is arrhythmic in DD. This suggests that the sLNv function in sustenance of activity rhythms is dependent not only on PDF oscillations in its DP, but on additional mechanisms that are independent of oscillating PDF. Previous studies showed that breakdown of behavioural rhythms was accompanied by loss of PDF oscillations in sLNv DP without affecting sLNv molecular oscillations [16,17,24], while in our study behavioural arrhythmicity is accompanied by loss of sLNv PER without loss of PDF oscillations. In other words, PDF oscillations in sLNv DP are necessary, but in the absence of functional sLNv clocks, they are not sufficient for behavioural rhythmicity. We show for the first time that oscillating PDF in sLNv DP and synchronous molecular clocks in PDF^-^neurons is not accompanied by rhythmic locomotor activity. In the prior studies the strong association of behavioural rhythmicity with PDF oscillations in sLNv DP, led to an implicit assumption of causality and hence, a need to invoke an additional component in sLNv did not arise. In *pdf>Q128*, due to disconnect of PDF oscillations in sLNv DP from behavioural rhythms, we have been able to uncover the possible existence of a potential additional mechanism in sLNv in mediating rhythmic behaviour in DD. We refer to this as the PDF oscillation independent component (POIC). We propose that both components of sLNv function: oscillations of PDF in DP acting as a synchronising agent of molecular oscillations in PDF^-^ neurons and POIC are required for coherent and robust activity rhythms in DD (Left panel in Fig 6).

**POIC**: PER-dependent molecular clock and PDF in sLNv soma are likely integral components of the POIC since, in *pdf>Q128* behavioural arrhythmicity is associated with loss of both. There is some indirect evidence for the presence of POIC in sLNv. The sufficiency of PER expression in sLNv in a *per*^*0*^ background rescuing behavioural arrhythmicity in DD and molecular oscillations in sLNv is well established [4,39,40]. Rescue of activity rhythms of *per*^*0*^ by restoring PER oscillations in sLNv was shown to be via a direct effect of PDF through PDFR on TIM levels in PDF^-^ circadian neurons [40]. However, this behavioural rhythm rescue did not involve the restoration of molecular clocks in PDF^-^ neurons. This suggests a role for sLNv in sustenance of free-running activity rhythms that is dependent on sLNv molecular clock and directly mediated by PDF but independent of PDF oscillations in DP and synchronous molecular clocks in PDF^-^ neurons [40]. A recent study showed that in the absence of PDF in sLNv DP, presence of PDF in sLNv soma and functional clocks in sLNv, cannot induce behavioural rhythmicity in DD [41], suggesting that the POIC alone cannot sustain rhythmicity. The relative contributions of the two components in sustaining rhythmicity need to be evaluated. Physiological roles of sLNv are not limited to oscillating PDF; sLNv functional output in the form of rhythmic changes in electrical activity, structural plasticity such as axonal morphology and pre-synaptic active sites may also contribute to behavioural rhythmicity [42,43]. Hyperactivating LNv in a *pdf*^*01*^ background, results in short period free-running rhythms, providing evidence for PDF-independent rhythms [20].

POIC could potentially be involved in conveying information from sLNv to motor centres to drive rhythmic behaviour in DD (Fig 6 left). sLNv have been hypothesised to directly modulate pre-motor centres to bring about rhythmic behaviour [14]. sLNv arbours form synaptic contacts in a time of day dependent manner with specific clusters of mushroom body and neurons of Pars Intercerebralis, a locomotor centre shown to control rhythmic activity behaviour [37,43] and such time dependent contacts could potentially involve POIC. sLNv modulates Leukokinin neurons and Leukokinin neuronal communication with its receptor neurons are a critical output circuit for rhythmic locomotor activity in DD [44]. sLNv can also indirectly communicate with motor centres: via its synaptic arbours with DN1p, which in turn synapse with a subset of PI neurons that are critical for rhythmic behaviour in DD [35,37,40].

### Role of sLNv for activity rhythms in LD

A previous report showed reduction in M-anticipation on partial reduction of PDF (63%) only in sLNv soma, but control-like M-anticipation upon near complete reduction of PDF from sLNv soma and DP and incomplete reduction (44%) in lLNv [27]. Further, flies lacking PDF from sLNv soma [26] or flies lacking PDF only from sLNv DP [41] still show M-anticipation to LD. Using a combination of genetic and environmental manipulations, we create a phenotype with a specific and complete loss of PDF from sLNv (soma and DP) and now find that M-peak, M-anticipation and E-peak phase are unaffected under LD, showing that PDF from sLNv is not necessary for this behaviour. It has also been shown that PDF from lLNv is not necessary, whereas downregulation in both subsets alters activity rhythms under LD [27]. Therefore, we conclude that PDF from either sLNv or lLNv is sufficient for M-peak and M-anticipation (Right panel in Fig 6). In *pdf>Q128* flies, despite loss of PDF from sLNv, PDF from lLNv can bring about M-anticipation. A recent study provides evidence for synaptic connections between lLNv and LNd and provides a possible means of direct communication to modulate LD behaviour [38]. Our studies show that cyclic secretion of PDF from sLNv is dispensable for LD behaviour. This is in agreement with a previous report where flies entrain to LD despite constitutively high PDF in sLNv projections [45]. In conclusion, under LD, PDF from sLNv is not required for M-anticipation as long as PDF is available from lLNv.

PDF has been shown to be important for early development of sLNv circuit [46]. Since in our study, complete loss of sLNv PDF was established at a late age of 23d, we cannot rule out the importance of PDF at younger ages of adulthood. It is possible that after the circuits have been established and mature, PDF in sLNv is dispensable for LD behaviour.

Rescue of PER in sLNv in *per*^*0*^ flies was sufficient for M-activity and anticipation in LD, suggesting that for M-anticipation, functional clocks in sLNv are essential [39]. However, rescuing PER in PDF^-^ neurons was also sufficient for M-anticipation [5], suggesting that PER in LNv is not necessary for M-activity in the presence of functional clocks in PDF^-^ neurons. But, this was hypothesised to occur via recovery of PDF output function of LNv [5]. We find that in LD, flies show morning anticipation despite the loss of PER in LNv and with complete loss of PDF from sLNv, providing evidence that persistence of M-anticipation in flies without functional LNv clocks occurs without recovering sLNv PDF output function as was suggested previously [5]. Thus, we conclude that molecular clocks in sLNv are not necessary for activity rhythms in LD.

### Role of soma clock for rhythmic PDF oscillations in sLNv DP

Expression of HTT-Q128 in LNv leads to loss of PER and PDF loss only from sLNv soma. Even in a mouse model of HD (*R6*/2), core clock gene expression in the Supra Chiasmatic Nucleus is attenuated and does not oscillate [47,48]. In a circadian fly model of another polyglutamine disorder, the Machado Joseph Disease, loss of PER from pacemaker neurons was reported [49]. Wild-type human Huntingtin has been shown to be involved in various stages of gene expression such as transcription, transport of mRNAs and translation [50]. Pathogenic Huntingtin has been implicated in transcriptional dysregulation either by sequestering critical players of transcriptional machinery, epigenetic modifications of chromatin or by directly binding to DNA [50]. Loss of PER from LNv could be a result of HTT-Q128 induced transcriptional dysregulation leading to downregulation of circadian gene expression.

We observe that, despite expression of HTT-Q128 in both the LNv subsets, PDF is selectively lost from sLNv soma, while PDF in lLNv is unaffected. Such specificity for sLNv was reported for the Machado Joseph disease model where only PDF^+^ sLNv numbers diminished [49]. Selective vulnerability of neuronal subsets is a hallmark of HD [51] and the selective susceptibility of sLNv to HTT-Q128 could be due to several factors such as its early development, size, and enrichment of neurotoxic factors or impoverishment of neuroprotective factors. Our observation that in sLNv, PDF is lost from the soma alone, while being detected in DP is quite unique. This is because, so far, in models of neurological disorders, selectivity for soma has not been reported; in fact, in many cases the axonal degeneration precedes loss of soma [51,52]. Even in fly models, expression of neurodegenerative proteins in *Drosophila* circadian neurons result in a decline of PDF signals in sLNv DP or abnormal sLNv axonal arborisations, while PDF in soma is unaffected [53–56]. A possible explanation for the detrimental effect of pathogenic Huntingtin on sLNv soma over axonal termini is the differential distribution of HTT-Q128 aggregates which are numerous in sLNv soma or in the region where sLNv soma are likely to be found, while aggregates are fewer and far apart in their axons and might not be sufficient to disrupt PDF in the axons.

We find it intriguing that PDF oscillations persist in sLNv DP despite loss of PER, a key molecular clock component and PDF from sLNv soma. We interpret these PDF oscillations to be indicative of rhythmic secretion of PDF since the functional consequence to them in the form of synchronous oscillations in PER levels between PDF^-^ neurons persists. Pathogenic Huntingtin is known to block axonal transport [28]. In *pdf>Q128* the oscillations in PDF levels at sLNv axonal termini suggests that either axonal transport in these flies is not impaired, or any impairment does not disrupt PDF oscillations in sLNv. The persistence of PDF oscillations in DP even in the absence of PDF in sLNv soma (Fig 3), and despite overexpression of the *pdf* gene in LNv [57], suggests that cyclic accumulation of PDF in sLNv terminals does not depend on levels of PDF in sLNv soma. However, since we find that pathogenic Huntingtin does not affect PDF in lLNv, these cells could potentially be responsible for driving PDF oscillations in sLNv DP. In *per* and *tim* null mutants, PDF mRNA shows oscillations in the soma of sLNv, although PDF peptide oscillations in sLNv DP was lost, suggesting post transcriptional clock control of PDF [12,58]. When PDF is ectopically expressed, rhythmic accumulation occurs only in projections of pacemaker sLNv, but not in non-circadian neurons, providing further evidence for clock control of PDF rhythms [57]. This posttranscriptional circadian regulation of PDF could take place at the level of peptide processing, axonal transport to terminals, accumulation or secretion [12,57]. However, so far, there is no direct evidence suggesting that PDF oscillations in sLNv DP are controlled by PER-driven clocks of sLNv. In pathogenic Huntingtin expressing flies, we are mostly unable to detect PER in sLNv soma and we conclude that PER is lost from sLNv. We concede that PER might be present below detection levels; but, that at such low levels unlikely to oscillate and might amount to loss of robust PER-driven molecular clock. We find that under DD, PER oscillations are lost even in lLNv and therefore, lLNv PER is unlikely to contribute to PDF oscillations in sLNv DP. Our results of continued PDF oscillations in sLNv DP in the absence of PER in sLNv soma in DD suggests involvement of PER-independent clock mechanisms in sLNv in mediating these oscillations. Other clock proteins involved in the second feedback loop such as Vrille and PDP1 could oscillate and this may suffice to induce PDF oscillations in sLNv DP [59]. An alternative to PER-driven clocks are the rhythms of oxidation-reduction of peroxiredoxins that persists in *Drosophila* circadian clock mutants, albeit with an altered phase [60]. We show an apparent independence of PDF oscillations in sLNv DP from PER-driven clocks in sLNv, thus, prompting a need to investigate alternate sources driving these oscillations in critical pacemaker neurons.

## Supporting information

S1 Fig Rhythmic and arrhythmic *pdf>Q128* flies show a similar distribution of PDF^+^ sLNv soma. (a) Frequency distribution of proportion of brain samples with 0 or more PDF^+^ soma (sLNv or lLNv) at each age is plotted for rhythmic *pdf>Q0* and *pdf>Q128* that are rhythmic and arrhythmic. The distribution of *pdf>Q0* is significantly different from rhythmic *pdf>Q128* (blue *) and from arrhythmic *pdf>Q128* (red *). * *p*<0.005 and *** *p*<0.001. (b) Mean LNv soma numbers (top) and signal intensity of PDF in them (bottom) for *pdf>Q128* and *pdf>Q0* for 6d old flies under LD. * indicates difference between genotypes at *p*<0.0001. (c) Percentage rhythmicity of flies is plotted where *indicates significant difference from the controls which have close to 100% rhythmicity at *p*<0.0001. (d) Top: Representative images of 9d old brains of *pdf>Q128* and *pdf>Q128*,*PDF* stained for PDF (green) and HTT (red) showing lLNv soma (arrowheads). Scale bars are 10 μm. Bottom: Mean number of PDF^+^ sLNv and lLNv soma per hemisphere for the two genotypes. Mostly no PDF^+^ sLNv soma is detectable in both the genotypes. Across panels, error bars are SEM.
(PDF)

S2 Fig Pathogenic Huntingtin expressing flies show a loss of sLNv PER and a reduction in lLNv PER under DD. (a) sLNv soma in 9d old flies in DD at CT23. Left: Frequency distribution of the proportion of hemispheres with 0 to 5 PER^+^ sLNv soma in *pdf>Q128* and *pdf>Q0*. *** indicate significantly differing distributions at *p*<0.001. Middle: Mean number of PDF^+^ or PER^+^ sLNv soma in *pdf>Q128* and *pdf>Q0* in DD. *** indicate statistically significant differences between genotypes at *p<0*.*001*: in magenta for PDF^+^ sLNv and in green for PER^+^ sLNv. Right: Frequency distribution of the proportion of hemispheres staining 0 to 5 sLNv soma that are PDF^+^or PER^+^ for *pdf>Q128* flies. (b) Representative images of 9d old brains of *pdf>Q128* stained for PER (green) and PDF (magenta) illustrating a lack of PER from LNv even upon increasing the antibody concentration four-fold. Scale bars are 10 μm. (c) For lLNv soma. All other details are same as above. Left: ** indicate significantly different distributions at *p*<0.005. Middle: *** (in black) difference in numbers of lLNv soma that are PDF^+^ and PER^+^ at *p*<0.001. Right: *** indicate that the two distributions differ significantly at *p*<0.001.
(PDF)

S3 Fig Flies expressing pathogenic Huntingtin in LNv did not show altered activity/rest rhythms under LD. (a) Representative double plotted, normalized actograms for *pdf>Q128* and its controls under LD (~100 lux) for 25d (age 3d-27d) followed by DD. All other details are similar to Fig 5a. (b) The activity counts per 15min is plotted against zeitgeber time for *pdf>Q128* in comparison with either *pdf>Q0* (left) or *Q128* (centre) or *pdfGal* (right) for AW1 (top), AW2 (middle) and AW3 (bottom). All other details are similar to Fig 5b. (c-f) *pdf>Q128* is not different from its controls across AWs in terms of mean daytime activity counts per 12h, mean nighttime activity counts per 12h, morning anticipation index and evening anticipation index. Across panels, error bars are SEM.
(PDF)

S4 Fig Pathogenic Huntingtin expressing flies show loss of PER from sLNv even under LD. (a) sLNv soma in 6d old flies in LD at ZT23. Left: Frequency distribution of the proportion of hemispheres with 0 to 5 PER^+^ sLNv soma in *pdf>Q128* and *pdf>Q0*. *** indicate significantly differing distributions at *p*<0.001. Middle: Mean number of PDF^+^ or PER^+^ sLNv soma in *pdf>Q128* and *pdf>Q0* in DD. *** indicate statistically significant differences between genotypes at *p*<0.001: in magenta for PDF^+^ sLNv soma and in green for PER^+^ sLNv soma. Right: Frequency distribution of the proportion of hemispheres staining 0 to 5 sLNv soma that are PDF^+^ or PER^+^ for *pdf>Q128* flies. (b) For lLNv. All other details are same as above. Across panels, error bars are SEM.
(PDF)

S5 Fig Pathogenic Huntingtin expressing flies in LL show a loss of PDF from sLNv soma as well as DP. (a) Representative images of adult brains of *pdf>Q128* stained for PDF(green) and HTT (red) showing sLNv soma (arrows), lLNv soma (arrowheads), sLNv DP (triangles) and lLNv CP (double arrowheads) for LL (age 9d top, age 23d middle) and LD (age 23d bottom). Scale bars are 20 μm. Marked rectangles in each panel-set are enlarged in the subsequent panel. (b) Percentage of hemispheres with PDF in sLNv DP across age for *pdf>Q128* and *pdf>Q0* in LL. Symbols indicate statistically significant differences: # between genotypes at each age at *p*< 0.001 and * of *pdf>Q128* at age 16d from earlier ages and at 23d from earlier ages at *p*<0.01. (c) Top left: Mean number of sLNv soma per hemisphere in both regimes across age. At age 28d, flies in LL regime post age 23d have experienced 5d of LD. Symbols indicate statistically significant differences: * of LD-LD A28d from LD-LD A23d at *p*<0.01, # between regimes at specified age at *p*<0.001. Top right: Mean number of lLNv soma per hemisphere in both the regimes across age. ** indicate significant differences of age 28d from earlier ages in both regimes at *p*<0.001. Bottom left: Percentage of hemispheres with PDF in sLNv DP for both regimes across age. Symbols indicate statistically significant differences: *** of age 28d in LL-LD from earlier ages at *p*<0.0001 and ## between regimes at denoted ages at *p*<0.0001. Bottom right: Percentage of hemispheres with PDF in lLNv CP is plotted for both regimes across age. n = 20–24 hemispheres/genotype/age/regime. Across panels, error bars are SEM.
(PDF)

S1 Table The extent of variation in PER intensity within a neuronal group in pathogenic Huntingtin expressing flies is not higher than controls. Table shows within-neuronal group mean standard deviation in PER intensity for LNd and DN1 across time-points in DD for *pdf>Q128* and its control *pdf>Q0*. Standard deviation within LNd for *pdf>Q128* is significantly lower than control at CT23, CT5 and higher only at CT11, when in *pdf>Q0* PER is undetectable. Standard deviation within DN1 for *pdf>Q128* is significantly lower than *pdf>Q0* at CT23. *p<0.05, **p<0.01, ***p<0.001.
(PDF)

S1 Methods Image acquisition and analysis. (PDF)
