# Supplementary figures and images for: Oscillating PDF in termini of circadian pacemaker neurons and synchronous molecular clocks in downstream neurons are not sufficient for sustenance of activity rhythms in constant darkness
Source: PLoS One. 2017 May 30;12(5):e0175073. doi: 10.1371/journal.pone.0175073 (PMC5448722; doi:10.1371/journal.pone.0175073)

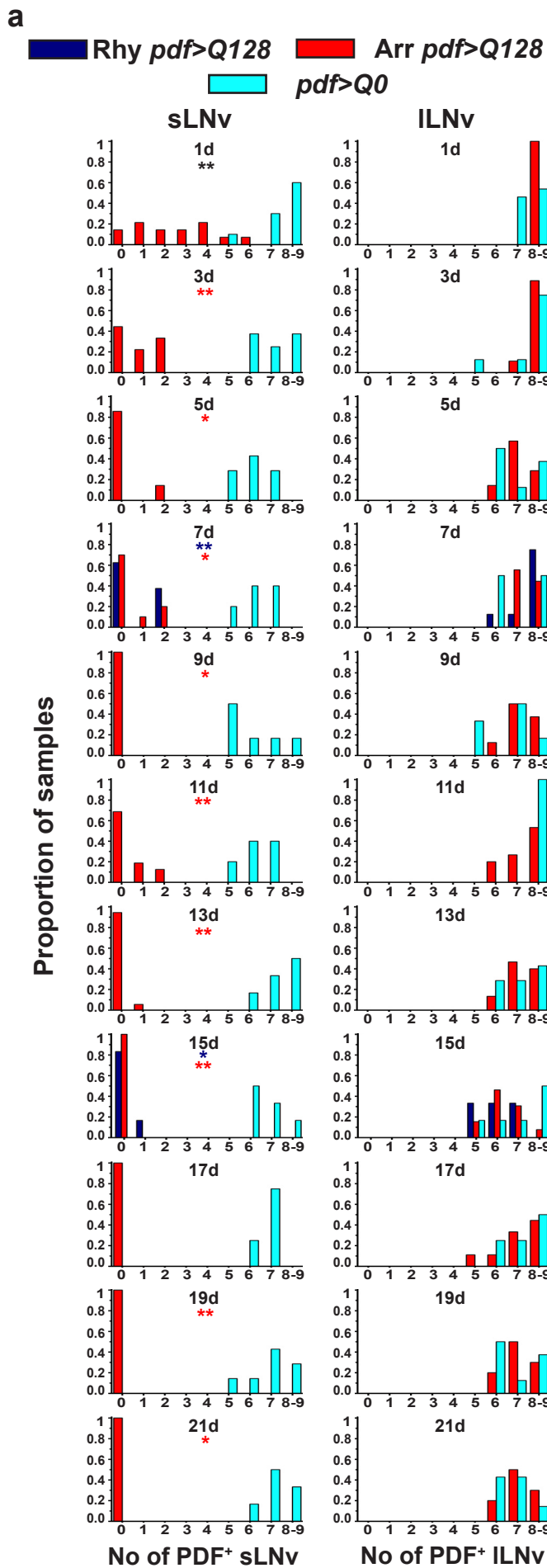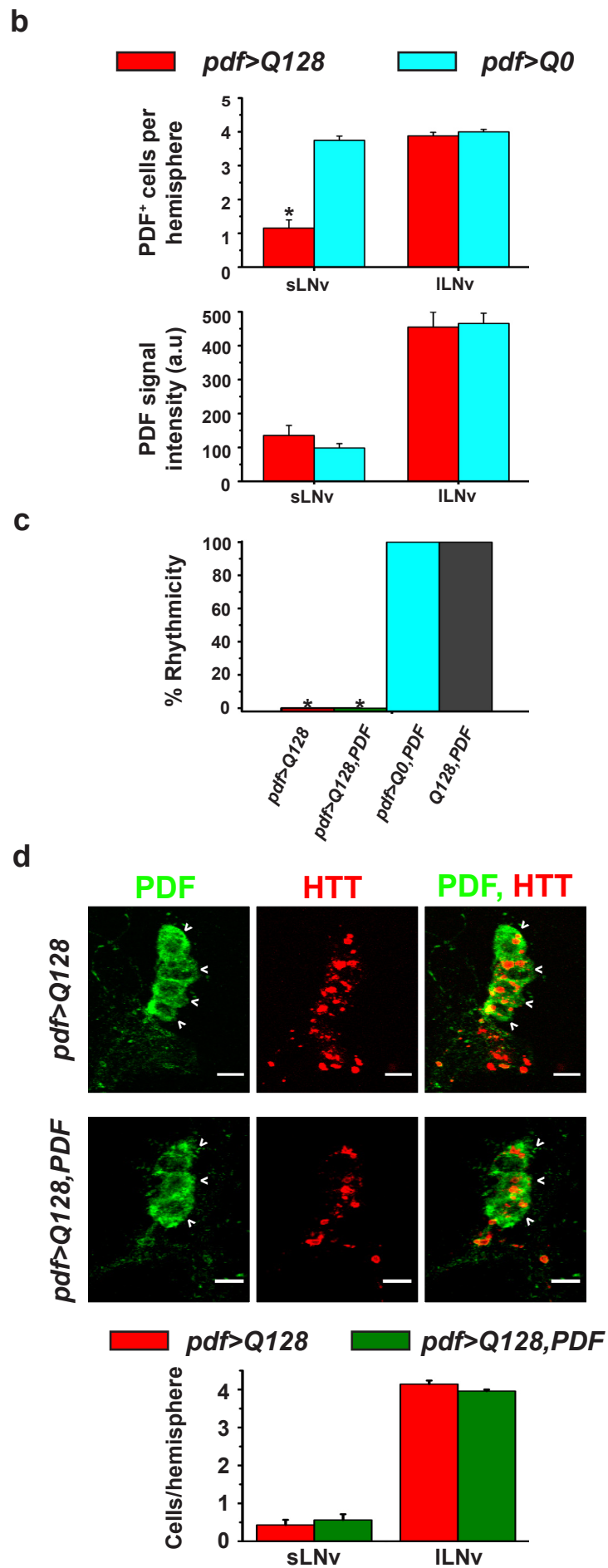

Supplement: S1 Fig — (a) Frequency distribution of proportion of brain samples with 0 or more PDF+ soma (sLNv or lLNv) at each age is plotted for rhythmic pdf>Q0 and pdf>Q128 that are rhythmic and arrhythmic. The distribution of pdf>Q0 is significantly different from rhythmic pdf>Q128 (blue *) and from arrhythmic pdf>Q128 (red *). * p<0.005 and *** p<0.001. (b) Mean LNv soma numbers (top) and signal intensity of PDF in them (bottom) for pdf>Q128 and pdf>Q0 for 6d old flies under LD. * indicates difference between genotypes at p<0.0001. (c) Percentage rhythmicity of flies is plotted where *indicates significant difference from the controls which have close to 100% rhythmicity at p<0.0001. (d) Top: Representative images of 9d old brains of pdf>Q128 and pdf>Q128,PDF stained for PDF (green) and HTT (red) showing lLNv soma (arrowheads). Scale bars are 10 μm. Bottom: Mean number of PDF+ sLNv and lLNv soma per hemisphere for the two genotypes. Mostly no PDF+ sLNv soma is detectable in both the genotypes. Across panels, error bars are SEM. (PDF) [file pone.0175073.s001.pdf]

■ *pdf>Q128*
■ *pdf>Q0*

■ PDF<sup>+</sup>
■ PER<sup>+</sup>

**a**

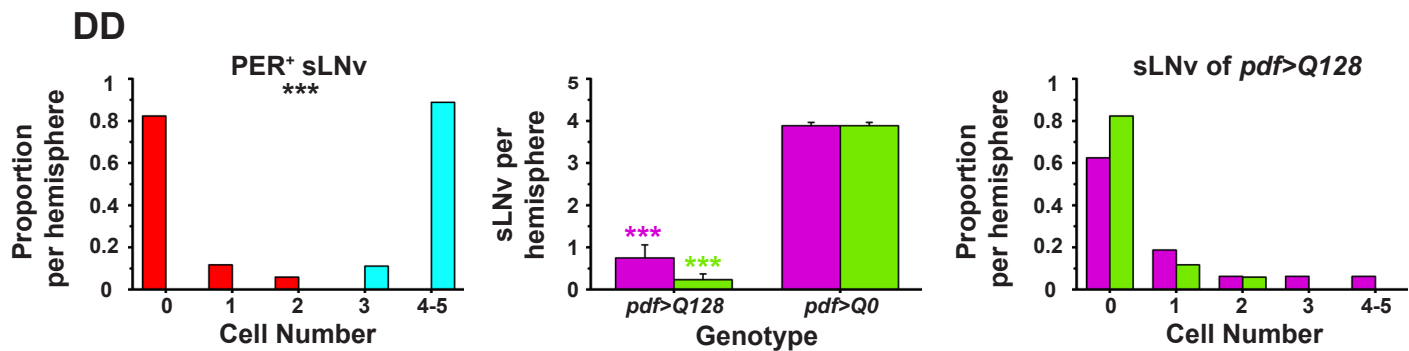

**b**

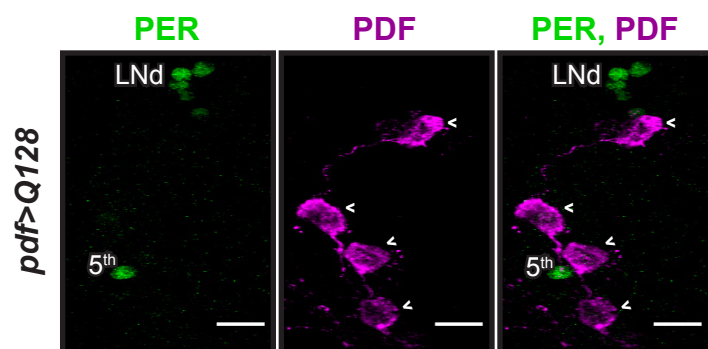

**c**

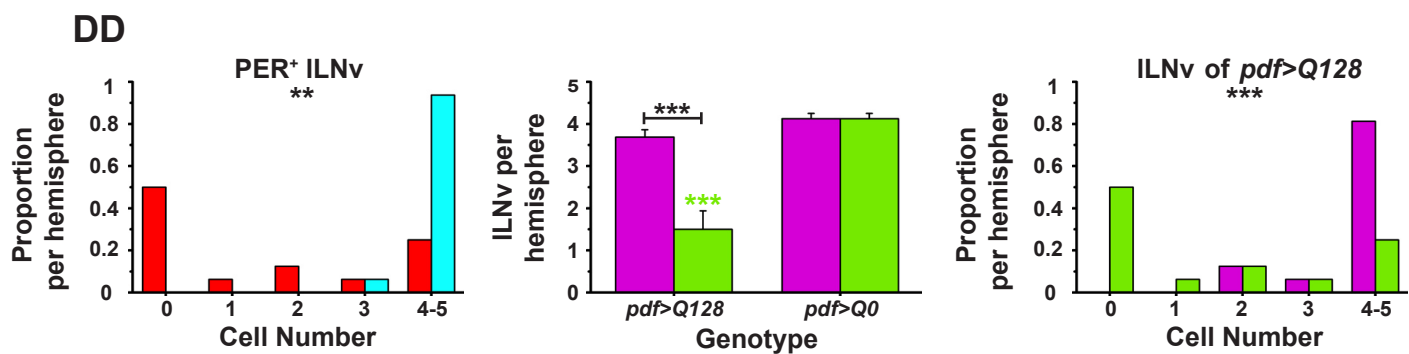

Supplement: S2 Fig — (a) sLNv soma in 9d old flies in DD at CT23. Left: Frequency distribution of the proportion of hemispheres with 0 to 5 PER+ sLNv soma in pdf>Q128 and pdf>Q0. *** indicate significantly differing distributions at p<0.001. Middle: Mean number of PDF+ or PER+ sLNv soma in pdf>Q128 and pdf>Q0 in DD. *** indicate statistically significant differences between genotypes at p<0.001: in magenta for PDF+ sLNv and in green for PER+ sLNv. Right: Frequency distribution of the proportion of hemispheres staining 0 to 5 sLNv soma that are PDF+or PER+ for pdf>Q128 flies. (b) Representative images of 9d old brains of pdf>Q128 stained for PER (green) and PDF (magenta) illustrating a lack of PER from LNv even upon increasing the antibody concentration four-fold. Scale bars are 10 μm. (c) For lLNv soma. All other details are same as above. Left: ** indicate significantly different distributions at p<0.005. Middle: *** (in black) difference in numbers of lLNv soma that are PDF+ and PER+ at p<0.001. Right: *** indicate that the two distributions differ significantly at p<0.001. (PDF) [file pone.0175073.s002.pdf]

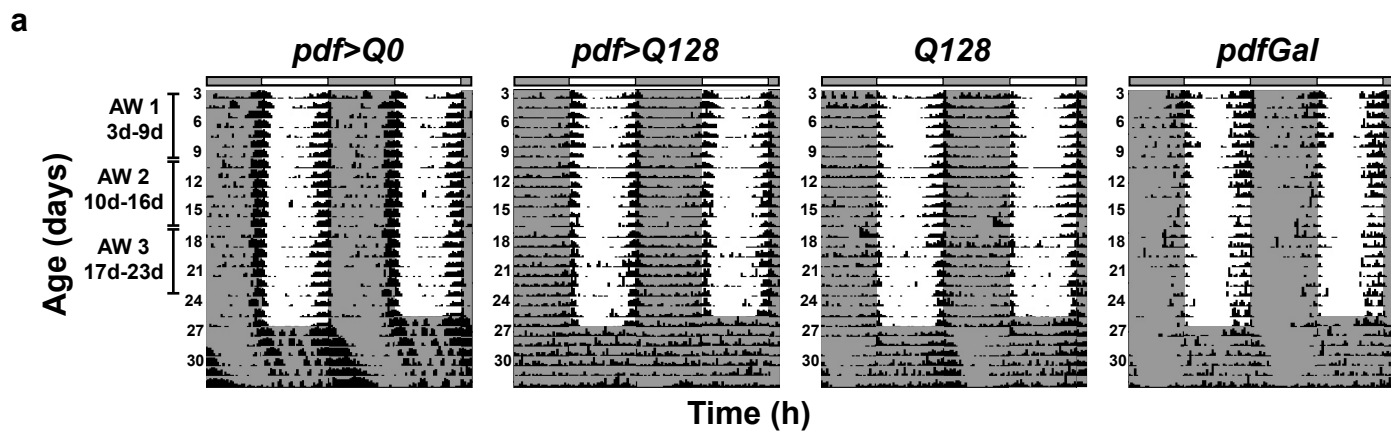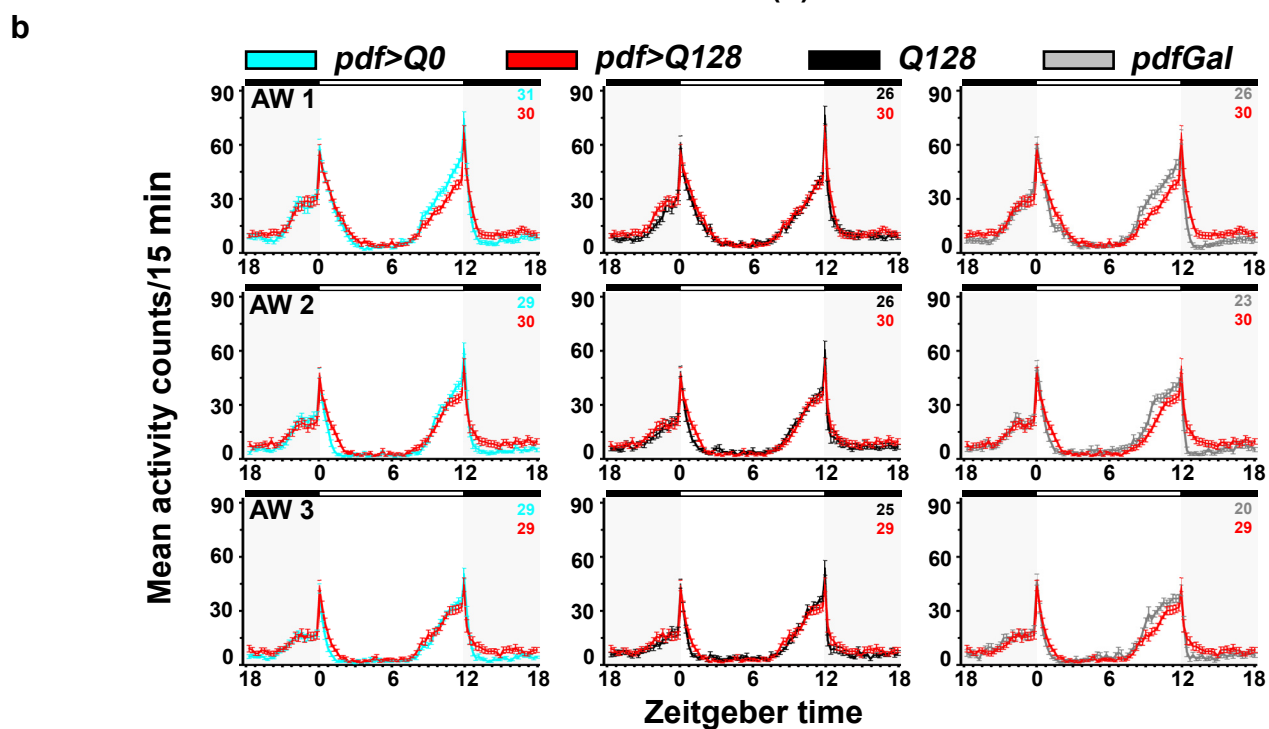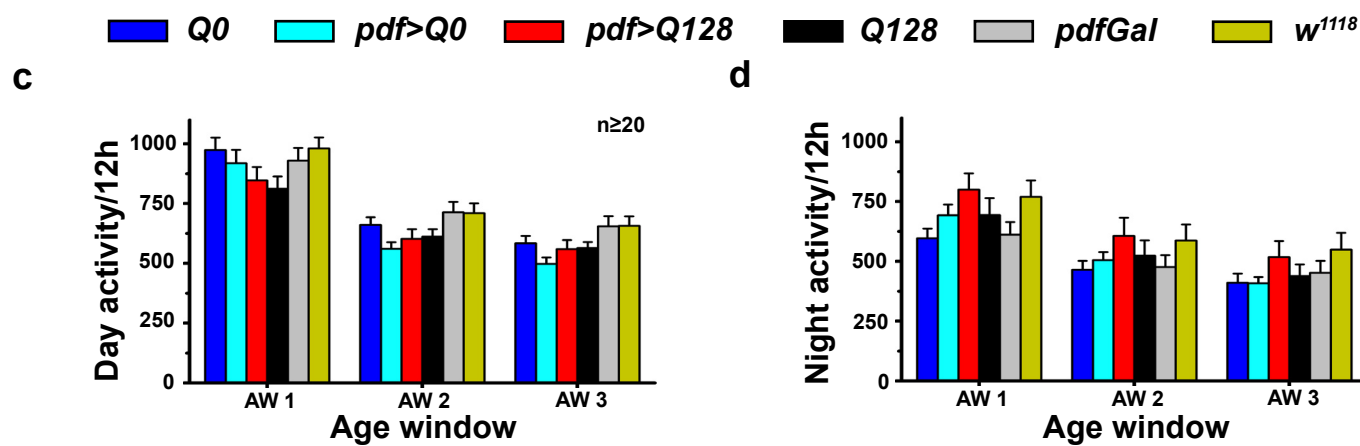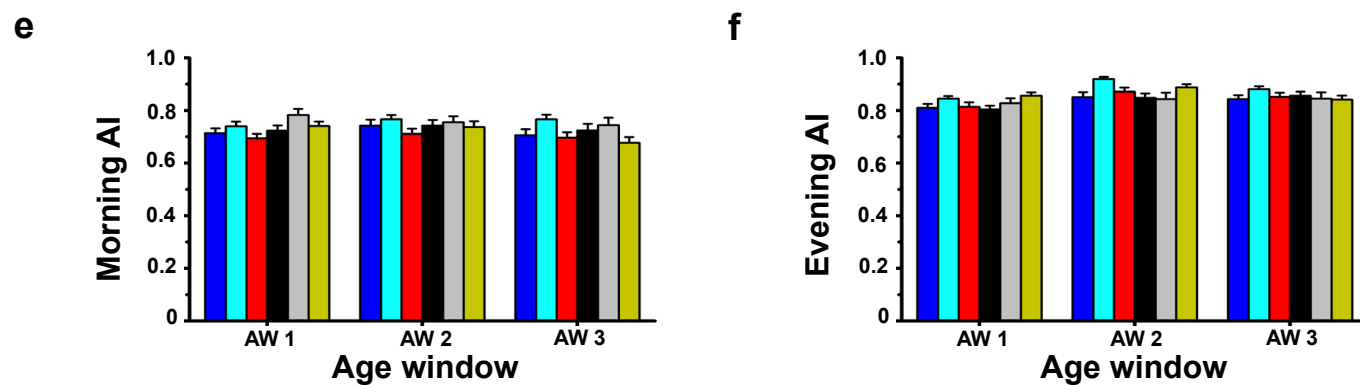

Supplement: S3 Fig — (a) Representative double plotted, normalized actograms for pdf>Q128 and its controls under LD (~100 lux) for 25d (age 3d-27d) followed by DD. All other details are similar to Fig 5a. (b) The activity counts per 15min is plotted against zeitgeber time for pdf>Q128 in comparison with either pdf>Q0 (left) or Q128 (centre) or pdfGal (right) for AW1 (top), AW2 (middle) and AW3 (bottom). All other details are similar to Fig 5b. (c-f) pdf>Q128 is not different from its controls across AWs in terms of mean daytime activity counts per 12h, mean nighttime activity counts per 12h, morning anticipation index and evening anticipation index. Across panels, error bars are SEM. (PDF) [file pone.0175073.s003.pdf]

pdf>Q128 pdf>Q0

PDF<sup>+</sup> PER<sup>+</sup>

a

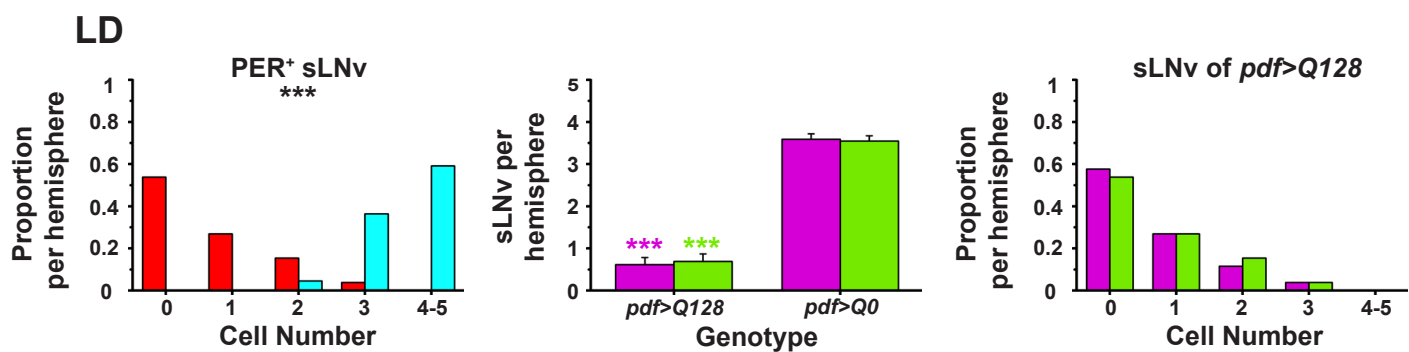

b

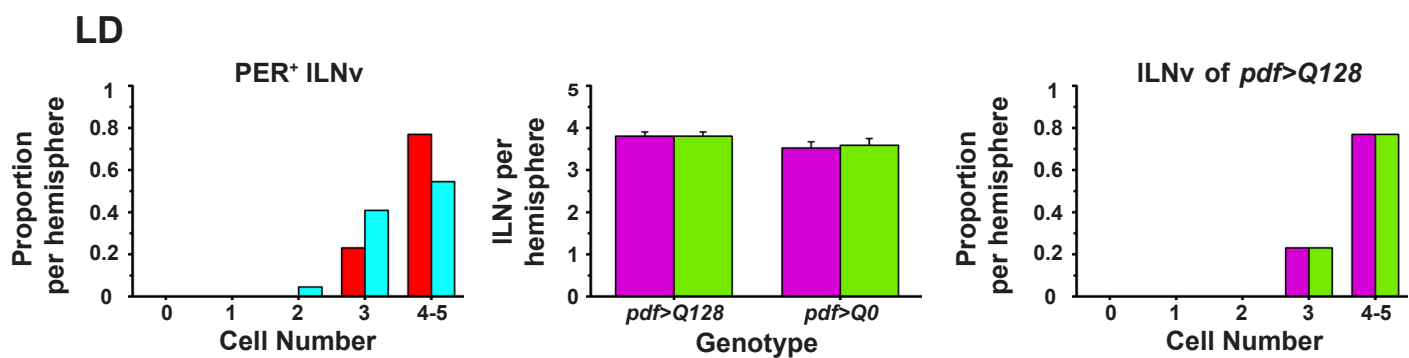

Supplement: S4 Fig — (a) sLNv soma in 6d old flies in LD at ZT23. Left: Frequency distribution of the proportion of hemispheres with 0 to 5 PER+ sLNv soma in pdf>Q128 and pdf>Q0. *** indicate significantly differing distributions at p<0.001. Middle: Mean number of PDF+ or PER+ sLNv soma in pdf>Q128 and pdf>Q0 in DD. *** indicate statistically significant differences between genotypes at p<0.001: in magenta for PDF+ sLNv soma and in green for PER+ sLNv soma. Right: Frequency distribution of the proportion of hemispheres staining 0 to 5 sLNv soma that are PDF+ or PER+ for pdf>Q128 flies. (b) For lLNv. All other details are same as above. Across panels, error bars are SEM. (PDF) [file pone.0175073.s004.pdf]
