# Supplementary material for: Oscillating PDF in termini of circadian pacemaker neurons and synchronous molecular clocks in downstream neurons are not sufficient for sustenance of activity rhythms in constant darkness
Source: PLoS One. 2017 May 30;12(5):e0175073. doi: 10.1371/journal.pone.0175073 (PMC5448722; doi:10.1371/journal.pone.0175073)
Supplement: S5 Fig — (a) Representative images of adult brains of pdf>Q128 stained for PDF(green) and HTT (red) showing sLNv soma (arrows), lLNv soma (arrowheads), sLNv DP (triangles) and lLNv CP (double arrowheads) for LL (age 9d top, age 23d middle) and LD (age 23d bottom). Scale bars are 20 μm. Marked rectangles in each panel-set are enlarged in the subsequent panel. (b) Percentage of hemispheres with PDF in sLNv DP across age for pdf>Q128 and pdf>Q0 in LL. Symbols indicate statistically significant differences: # between genotypes at each age at p< 0.001 and * of pdf>Q128 at age 16d from earlier ages and at 23d from earlier ages at p<0.01. (c) Top left: Mean number of sLNv soma per hemisphere in both regimes across age. At age 28d, flies in LL regime post age 23d have experienced 5d of LD. Symbols indicate statistically significant differences: * of LD-LD A28d from LD-LD A23d at p<0.01, # between regimes at specified age at p<0.001. Top right: Mean number of lLNv soma per hemisphere in both the regimes across age. ** indicate significant differences of age 28d from earlier ages in both regimes at p<0.001. Bottom left: Percentage of hemispheres with PDF in sLNv DP for both regimes across age. Symbols indicate statistically significant differences: *** of age 28d in LL-LD from earlier ages at p<0.0001 and ## between regimes at denoted ages at p<0.0001. Bottom right: Percentage of hemispheres with PDF in lLNv CP is plotted for both regimes across age. n = 20–24 hemispheres/genotype/age/regime. Across panels, error bars are SEM. (PDF) [file pone.0175073.s005.pdf]

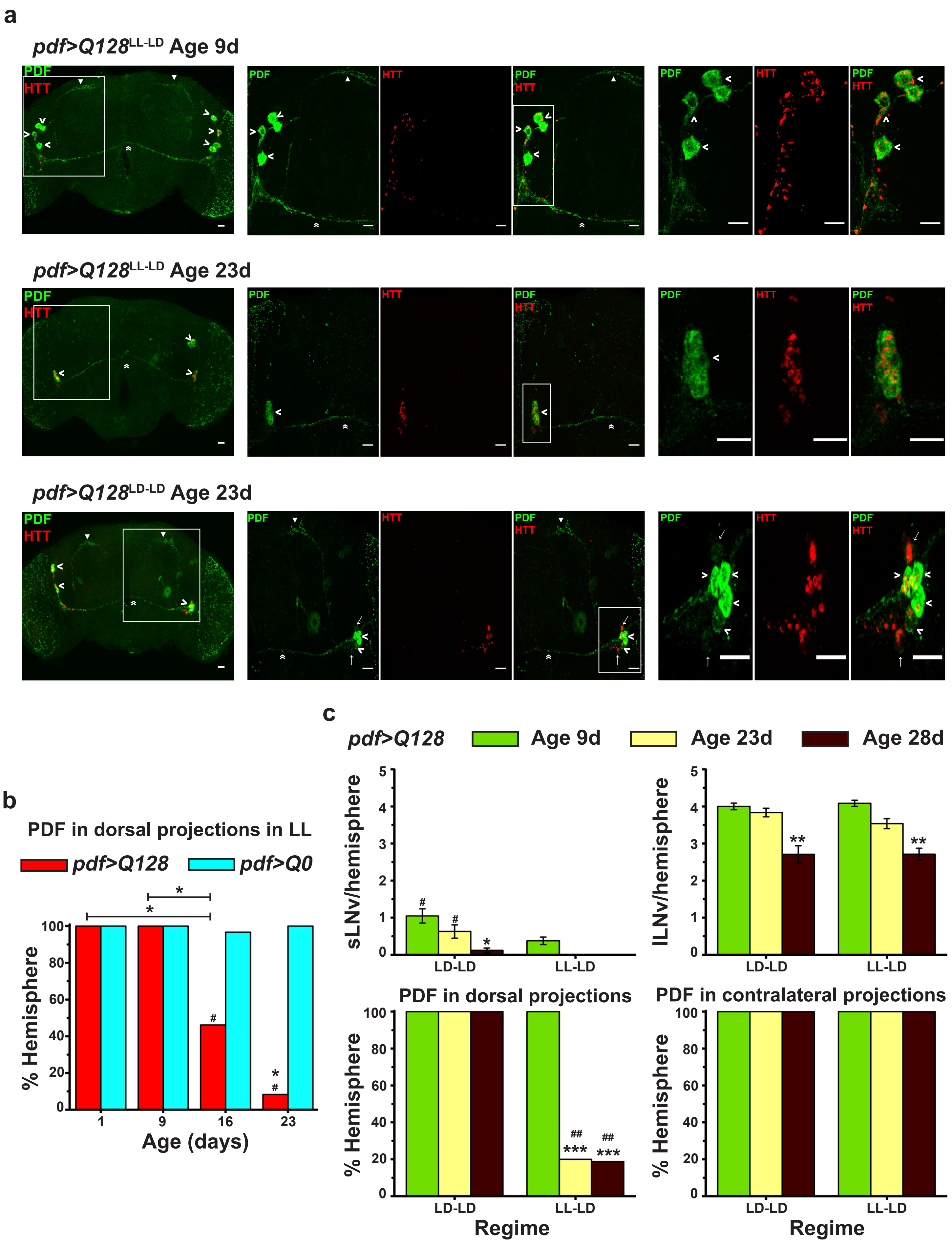
a
pdf>Q128LL-LD Age 9d
PDF
HTT
pdf>Q128LL-LD Age 23d
pdf>Q128LD-LD Age 23d
b
PDF in dorsal projections in LL
pdf>Q128
pdf>Q0
% Hemisphere
Age (days)
1
9
16
23
0
20
40
60
80
100
*
#
c
pdf>Q128
Age 9d
Age 23d
Age 28d
sLNv/hemisphere
lLNv/hemisphere
LD-LD
LL-LD
PDF in dorsal projections
PDF in contralateral projections
% Hemisphere
Regime
**
***
##
