# Supplementary material for: Oscillating PDF in termini of circadian pacemaker neurons and synchronous molecular clocks in downstream neurons are not sufficient for sustenance of activity rhythms in constant darkness
Source: PLoS One. 2017 May 30;12(5):e0175073. doi: 10.1371/journal.pone.0175073 (PMC5448722; doi:10.1371/journal.pone.0175073)
Supplement: S1 Table — Table shows within-neuronal group mean standard deviation in PER intensity for LNd and DN1 across time-points in DD for pdf>Q128 and its control pdf>Q0. Standard deviation within LNd for pdf>Q128 is significantly lower than control at CT23, CT5 and higher only at CT11, when in pdf>Q0 PER is undetectable. Standard deviation within DN1 for pdf>Q128 is significantly lower than pdf>Q0 at CT23. *p<0.05, **p<0.01, ***p<0.001. (PDF) [file pone.0175073.s006.pdf]

LNd		
	pdf>Q128	*pdf>Q0*
**CT23**	102.81±28.21	132.15±16.6**
**CT5**	80.6±7.44	116.91±9.26**
**CT11**	34.41±8.73***	0
**CT17**	113.45±12.9	124.26±10.69
**DN1**		
**CT23**	79.93±7.13	99.98±9.08 *
**CT5**	58.85±3.04	55.97±3.77
**CT11**	40.58±3.73	44.76±3.46
**CT17**	56.38±5.81	57.93±3.4
