# Supplementary material for: Oscillating PDF in termini of circadian pacemaker neurons and synchronous molecular clocks in downstream neurons are not sufficient for sustenance of activity rhythms in constant darkness
Source: PLoS One. 2017 May 30;12(5):e0175073. doi: 10.1371/journal.pone.0175073 (PMC5448722; doi:10.1371/journal.pone.0175073)
Supplement: S1 Methods — (PDF) [file pone.0175073.s007.pdf]

S1 Methods

Image Acquisition and Analysis

The samples were viewed under a Zeiss Axio Observer Z1 epifluorescence microscope using the 63X/oil1.4 objective and PDF^+ LNv were counted and the presence of DP was noted. For quantification of PDF intensity in sLNv DP, images were captured as a z-stack of 1µm interval using the 40X/oil1.3 objective, keeping the lamp intensity and exposure time constant across samples. NIH imaging software ImageJ was used on maximum intensity projection background subtracted images to quantify PDF intensity in DP by subtracting DP intensity from area matched background by an analyser blind to the genotype and time-point. To confirm oscillations of PDF in sLNv DP of *pdf>Q128*, at least 3 independent experiments per light regime with two time-points corresponding to the peak and trough were carried out. For quantification of PER intensity, images were captured as described above. Using image J, the PER intensity for a cell at its sharpest z-plane was obtained and the average PER intensity of that group was subtracted from an equal area of average background intensity in the vicinity [1]. At ZT11, as no or negligible PER signal was observed in both genotypes, PER intensity was assigned a low value of 0.001. In DD also, when PER signal was not detected above the background on quantification, PER intensity was assigned 0.001. In DD, when a LNv was observed with PDF staining, but no corresponding signal was observed over the background in PER, the PER intensity for those LNv were assigned a value of 0.001. To determine the extent of synchrony within a neuronal group, the standard deviation (SD) for PER intensity was calculated for each hemisphere and the mean SD across hemispheres was compared between genotypes for each time-point [2]. For representative images, confocal z-stacks were captured using Zeiss LSM700, LSM 880 and Olympus FV1000. To aid

visualisation of DNs, they have been uniformly imaged at a higher laser power and PMT gain compared to LNs.

Statistical Analysis

For cell numbers, a 2-way ANOVA with genotype and age or regime and age as fixed factors was performed. For PDF and PER staining intensity, a 2-way factorial ANOVA was carried out with genotype and time as the fixed factors. Post hoc multiple comparisons were carried out using Tukey's Honest Significant Difference test at $\alpha = 0.05$. For cell number distributions, Kolmogorov-Smirnov test was done at $\alpha = 0.05$.

References

1. Sheeba V, Sharma VK, Gu H, Chou YT, O'Dowd DK, Holmes TC (2008c) Pigment dispersing factor-dependent and -independent circadian locomotor behavioral rhythms. J Neurosci 28: 217-227.
2. Zhang L, Chung BY, Lear BC, Kilman VL, Liu Y, Mahesh G, et al. (2010) DN1(p) circadian neurons coordinate acute light and PDF inputs to produce robust daily behavior in *Drosophila*. Curr Biol 20: 591-599.
